# Low Blue Dose Photodynamic Therapy with Porphyrin-Iron Oxide Nanoparticles Complexes: In Vitro Study on Human Melanoma Cells

**DOI:** 10.3390/pharmaceutics13122130

**Published:** 2021-12-10

**Authors:** Simona Nistorescu, Ana-Maria Udrea, Madalina Andreea Badea, Iulia Lungu, Mihai Boni, Tatiana Tozar, Florian Dumitrache, Valentin-Adrian Maraloiu, Roua Gabriela Popescu, Claudiu Fleaca, Ecaterina Andronescu, Anca Dinischiotu, Angela Staicu, Mihaela Balas

**Affiliations:** 1 National Institute of Laser, Plasma and Radiation Physics, 409 Atomistilor Str., 077125 Magurele, Romania; simona.stroescu@inflpr.ro (S.N.); ana.udrea@inflpr.ro (A.-M.U.); iulia.lungu@inflpr.ro (I.L.); mihai.boni@inflpr.ro (M.B.); tatiana.alexandru@inflpr.ro (T.T.); florian.dumitrache@inflpr.ro (F.D.); claudiu.fleaca@inflpr.ro (C.F.); 2 Department of Biochemistry and Molecular Biology, Faculty of Biology, University of Bucharest, 91-95 Splaiul Independentei, 050095 Bucharest, Romania; badea_andreea08@yahoo.com (M.A.B.); roua.popescu@drd.unibuc.ro (R.G.P.); ancadinischiotu@yahoo.com (A.D.); 3 Research Institute of the University of Bucharest, Earth, Environmental and Life Sciences, Section-ICUB, 050663 Bucharest, Romania; 4 Faculty of Applied Chemistry and Materials Science, University Politehnica of Bucharest, 1-7 Gh. Polizu Str., 011061 Bucharest, Romania; ecaterina.andronescu@upb.ro; 5 National Institute of Materials Physics, 405 A Atomistilor Str., 077125 Magurele, Romania; maraloiu@infim.ro

**Keywords:** photodynamic therapy PDT, porphyrin, iron oxide nanoparticles, melanoma

## Abstract

The purpose of this study was to investigate the effectiveness in photodynamic therapy of iron oxide nanoparticles (γ-Fe_2_O_3_ NPs), synthesized by laser pyrolysis technique, functionalized with 5,10,15,20-(Tetra-4-sulfonatophenyl) porphyrin tetraammonium (TPPS) on human cutaneous melanoma cells, after only 1 min blue light exposure. The efficiency of porphyrin loading on the iron oxide nanocarriers was estimated by using absorption and FTIR spectroscopy. The singlet oxygen yield was determined via transient characteristics of singlet oxygen phosphorescence at 1270 nm both for porphyrin functionalized nanoparticles and rose bengal used as standard. The irradiation was performed with a LED (405 nm, 1 mW/cm^2^) for 1 min after melanoma cells were treated with TPPS functionalized iron oxide nanoparticles (γ-Fe_2_O_3_ NPs_TPPS) and incubated for 24 h. Biological tests revealed a high anticancer effect of γ-Fe_2_O_3_ NPs_TPPS complexes indi-cated by the inhibition of tumor cell proliferation, reduction of cell adhesion, and induction of cell death through ROS generated by TPPS under light exposure. The biological assays were combined with the pharmacokinetic prediction of the porphyrin.

## 1. Introduction

Melanoma is the most lethal type of skin cancer and the third form of malignancy encountered between the ages of 15 to 39 years, with an incidence constantly increasing. Among the risk factors involved in the evolution of aggressive skin the malignant tumor are found: family history, ultraviolet (UV) radiation exposure, or simply variations in pigmentation genes [1,2,3]. The pathogenic mechanism of melanoma involves two cell subpopulations in the epidermis, called melanocytes and keratinocytes. As a result of UV exposure, skin keratinocytes increase melanin generation by producing the melanocyte-stimulating hormone (MSH) that binds the melanocortin receptor 1 (MC1R) found on melanocytes surface. Briefly, the melanocytes transfer melanin to surrounding keratinocytes and protect the living cells. The melanoma risk appears as a cause of long exposure of the nuclei to UV damage and accumulation of the mutations in sensitive regions [3,4,5,6,7].

For the treatment of melanoma, a lot of therapies were developed: chemotherapy, immunotherapy, surgical resection, biochemotherapy, photodynamic therapy, and targeted therapy [8]. Among these, photodynamic therapy (PDT) is based on the generation of reactive oxygen species (ROS), especially singlet oxygen, by light exposure of unhealthy tissue, at a specific wavelength. The most important aspect of the procedure is the use of a photosensitizer (PS), a non-toxic agent that accumulates in the tumor and generates ROS by light irradiation [9,10,11]. Briefly, the PS molecule is first excited to the singlet state and through intersystem crossing forms the triplet state that then transfers the energy to ground state oxygen, generating excited singlet state oxygen (^1^O_2_), the chief reactive species, which interacts with surrounding molecules and destroys them [9,12]. A PS agent must be: targeted to the tumor (able to accumulate in the tumor), a non-toxic compound, painless, easy to eliminate, stable in time, active only on illumination, and efficient in singlet oxygen generation [12,13].

Porphyrins, a group of organic compounds, are considered great PS agents that may be used to accept the energy of light and produce singlet oxygen in the presence of a triplet-state electron [9]. They are macrocyclic compounds with four pyrrole subunits interconnected at their α carbon atoms, are found in hemoglobin and cytochromes structure, and have the function of a cofactor for redox-active enzymes. Moreover, their applications in the biomedical field as PS in PDT are associated with their optical and redox properties [14,15]. A large range of porphyrin derivatives can be used in PDT. The sulfonation of tetraphenyl porphyrin generates the tetra sulfonate porphyrin, an excellent producer of singlet oxygen, with high solubility in water, and a promising PS for PDT. Also, tetra sulfonate porphyrin is permeable through the cell membrane and has specific accumulation and activity on tumor cells both in vitro and in vivo [14,15].

Generally, the PDT begins 24 h before the light exposure, when PS, such as porphyrin [10], is administrated for its deposition and accumulation in the tumor. Interestingly, it was described that PS effectiveness in blood at the irradiation time determines tumor vasculature damage and, finally, affects the nutrient supply of the tumor. Thus, the PS action is not concentrated only in the tumor, the effect of irradiation being not only the induction of tumor cell death [16,17,18,19,20,21,22]. Moreover, PDT presents the limitation of a long in vivo half-life of the PS and the difficulty of its transport in deep tumor regions of the body [23].

Nevertheless, the porphyrin activity as PS in the specific cell target can be improved by many carriers (nanoparticles, micelles), being avoided the photosensitivity on healthy tissue. Delivery systems protect the PS and release it only under specific conditions, where ROS generation is required [24,25].

The use of nanoparticles as drug delivery systems proved to have multiple advantages, such as: high biocompatibility, ease of fabrication and functionalization, and the control of material size, and shape. Iron oxide nanoparticles are considered important tools in the modulation of PS action due to their ability to carry and transport therapeutic amounts of PS in the deep tumor regions of the body and to enhance the solubility of hydrophobic PS [23]. Besides, the iron oxide nanoparticles present magnetic properties that can be manipulated for medical application (cell labeling, gene delivery) or hyper/photo-thermal therapy application. The iron oxide nanoparticles have been already used as vehicles for the delivery of PS in PDT [26,27].

In this context, the purpose of this study was to investigate the anti-tumor effectiveness of iron oxide nanoparticles (γ-Fe_2_O_3_ NPs) functionalized with 5,10,15,20-(Tetra-4-sulfonatophenyl) porphyrin tetraammonium (TPPS) on human cutaneous melanoma cells, after only 1 min light exposure. The water-soluble porphyrin, TPPS was chosen due to its potential application in cancer therapy, and infectious diseases [28].

The efficiency of porphyrin loading on the iron oxide nanocarriers, synthesized by laser pyrolysis technique, was estimated by using absorption and FTIR spectroscopy. Also, the singlet oxygen yield was determined via transient characteristics of singlet oxygen phosphorescence at 1270 nm for both, TPPS functionalized iron oxide nanoparticles (γ-Fe_2_O_3_ NPs_TPPS) and rose bengal used as standard. The irradiation was per-formed with a LED (405 nm, 1 mW) for 1 min after melanoma cells were treated with γ-Fe_2_O_3_ NPs_TPPS complexes and incubated for 24 hours. It was proved that the light exposure increased the production of ROS generated in melanoma cells by porphyrin loaded nanoparticles, reduced the proliferation and cellular adhesion capacity, and induced cell death.

## 2. Materials and Methods

### 2.1. Chemical Compounds

The photosensitizer, 5,10,15,20-(Tetra-4-sulfonatophenyl) porphyrin tetraammo-nium (TPPS) (purity > 95%), was purchased from PorphyChem SAS, Dijon, France. Rose Bengal (95% purity), used as PS standard, was bought from Sigma Aldrich (Darmstadt, Germany).

For iron oxide nanoparticles (NPs) synthesis by laser pyrolysis technique, the reactive gas-es/vapors used were iron pentacarbonyl (Fe(CO)_5_) (99.999%, Merck, Darmstadt, Germany) as an iron precursor, synthetic air (Linde, Dublin, Ireland) as an oxidizing agent, and ethylene (C_2_H_4_, 99.99%, Linde, Dublin, Ireland) as laser energy transfer agent (i.e., sensitizer).

### 2.2. Iron Oxide Nanoparticle Synthesis

**Figure 1:**
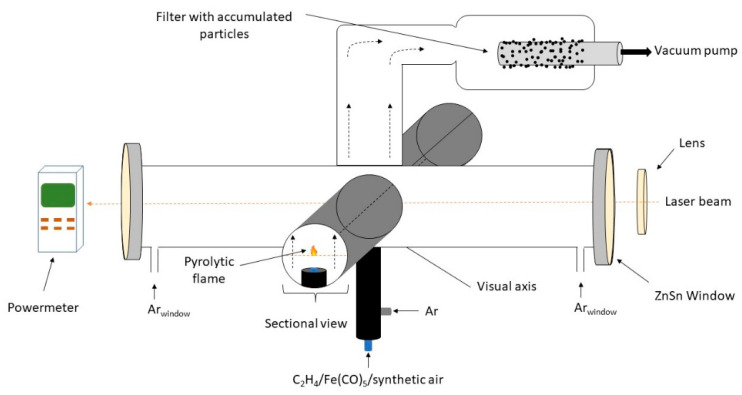
Laser pyrolysis of iron oxide nanoparticles.

The experimental set-up for laser pyrolysis technique is presented in Figure 1. Due to the resonance between the emission line of the CO_2_ infrared laser and the absorption line of the sensitizer gas, the ethylene molecules became vibrationally excited upon the interaction with IR photons, and the reactive mixture is heated very fast via collisions [29]. Also, the mixture is supplementary heated by the oxidation of iron-based species and ethylene molecules. Thus, the sudden formation of iron-based oxidized fragments/clusters is followed by their condensation into small iron oxide nanoparticles which emerge from the reaction zone.

As presented in Figure 1, the reactant flow orthogonally intersects the focused continuous beam emitted by the CO_2_ laser (70W maximum output power, λ = 10.6 µm). This reactant flow is comprised of a mix of synthetic air (33 sccm) and iron pentacarbonyl vapors (6.6 sccm) transported by a bubbled ethylene flow (33 sccm). Coaxial argon (Ar) flow is used to both confine the gas precursors to the flow axis, as well as to ‘guide’ the freshly nucleated nanoparticles towards the collection chamber. The reaction zone can be clearly identified through a flame that emits in the visible spectrum. The temperature of the flame needs to be constant throughout the experiment and it can be easily measured using an optical pyrometer. The process was described in detail elsewhere [29]. The experimental parameters used are presented in Table 1.

A water-soluble porphyrin, TPPS, was used as both a stabilizer and as an active component. Initially, the TPPS powder was added to a glass vessel with 20 mL distilled water (dH_2_O) having a pH = 5.5 and left 6 h in the ultrasound bath in order to ensure its solvation. Then the iron NPs were introduced in the TPPS solution, ensuring the equal concentration of 0.5 g/L of nanoparticles and TPPS. The 20 mL water-based suspension was homogenized in an ultrasound bath provided with a cooling system at 59 kHz and 20 °C for 12 h. The resulting suspension was then centrifuged at 10K rpm for 5 min and washed with dH_2_O several times, each time the solid deposit was redispersed while maintaining the initial water volume, with the ultrasonic bath (30 min).

### 2.3. Physicochemical Characterization

#### 2.3.1. Nanoparticle Characterization

In order to characterize the hydrodynamic size and stability of the nanoparticles suspensions, as well as the TPPS functionalized iron NPs suspensions, we utilized dynamic light scattering (DLS) measurements (Nanoparticle Analyzer SZ-100V2, Horiba, Kyoto, Japan) which employs a Diode-pumped solid-state (DPSS) laser emitting at 532 nm, with a power of 100–240 V AC ± 10%, 50 Hz/60 Hz; at a scattering angle of 173°. 1 mL volume of aqueous suspension was added in a quartz cuvette cell for hydrodynamic size measurements which were taken in triplicate for the following suspensions: γ-Fe_2_O_3_ (0.5 g/L) in dH_2_O, and γ-Fe_2_O_3_ (0.5 g/L) + TPPS (0.5 g/L) in dH_2_O. The same process was used for zeta potential measurements where a cell with electrodes (carbon, 6 mm) was used with a lower volume of solution.

XRD measurements on synthesized nanoparticles were carried out using a Panalytical X’Pert system (Malvern Panalytical Ltd., Malvern, United Kingdom) with Bragg-Bretano geometry: 0.02° step size, 30 s-time/step, 2θ: 15–80° with Cu Kα radiation tube and a graphite monochromator for the diffracted X-ray beam. The NPs from a diluted water suspension and also the final (stabilized and purified) water suspension containing NPs coated with TPPS was characterized with a transmission electron microscope (TEM) apparatus type Jeol ARM 200F also having an EDS facility. Two drops of water dispersions were deposed on a carbon-coated grid and allowed to dry at room temperature. The elemental composition of the as synthesized powder sample was evaluated by EDS-SEM using an FEI Quanta Inspect apparatus.

#### 2.3.2. Spectroscopic Methods

The efficiency of TPPS loading on iron oxide nanoparticles was determined by measuring the absorbance spectra using a Lambda 950 UV–Vis-NIR spectrophotometer (PerkinElmer, Inc., Waltham, MA, USA) and 10 mm thickness optical quartz cuvettes (PerkinElmer, Inc., Waltham, MA, USA). The absorption spectra were measured from 200 to 800 nm at room temperature (22–24 °C).

The experimental detection system used to measure the time-resolved phosphorescence signal of the photosensitized singlet oxygen at wavelength 1270 nm was described in detail elsewhere [30,31].

The laser beam used for excitation of TPPS molecules was emitted by the SHG (532 nm) of a Nd:YAG laser (6 ns pulse time width at half maximum, 10 Hz repetition rate, maximum energy per pulse 25 mJ) (Minilite II, Continuum, Excel Technology, Milpitas, CA, USA). The TPPS molecule is first excited to the singlet state and through intersystem crossing forms the triplet state and then transfers the energy to ground state oxygen, generating excited singlet state oxygen (^1^O_2_).

The laser spectroscopic technique for ^1^O_2_ species measurements is based on the radiative deexcitation of the singlet state through the phosphorescence emission at 1270 nm.

The phosphorescence is detected by a near-infrared (NIR) photomultiplier (PMT Module H10330, Hamamatsu, Japan) and measured with a digital scope (DPO-7254, Tektronix Inc, Beaverton, OR, USA). The phosphorescence transient signal is fitted with a mono-exponential function and the ^1^O_2_ lifetime is obtained is given by the decay constant.

The quantum yield of the singlet oxygen generation (ΦΔ) is determined using also a relative method to a reference standard. Rose Bengal (RB) which has 0.7 the quantum yield of singlet oxygen generation in water was used as a reference. The RB solutions and investigated solutions or suspensions were measured in the same experimental conditions and the quantum yield was determined using the equation [31]:

(1) $$\Phi_{\Delta }=\Phi_{\Delta }^{ref}\frac{I_{}}{I_{ref}}\frac{A_{ref}}{A}\frac{n_{}^{2}}{n_{ref}^{2}}\frac{\tau }{\tau_{ref}}$$

where Φ_Δ_ is the quantum yield of singlet oxygen generation, *I* is the singlet oxygen phosphorescence intensity at the starting time of the laser pulse for the measured sample, *A* is the optical absorption of the sample at the laser excitation wavelength (532 nm), *τ* is the lifetime of singlet oxygen in the measured sample and *n* is the solvent refractive index. The script *ref* indicates the measured values for the standard RB solutions. The intensity of phosphorescence at *t* = 0 was obtained by the extrapolation to zero of the mono-exponential fitting functions for the experimental decay curves.

IR spectra were recorded with a FTIR spectrometer, model Nicolet iS50, under transmission mode over the wavenumber range of 4000–400 cm^−1^, at a resolution of 4 cm^−1^. The TPPS solutions, the γ-Fe_2_O_3_ NPs, and complex γ-Fe_2_O_3_ NPs_TPPS suspensions were dried on optical grade KRS-5 plates.

The theoretical IR spectrum of TPPS was calculated using Gaussian09 software [32]. The molecular structure was subjected to geometry optimization followed by the calculation of vibrational wavenumbers using the density functional theory (DFT). The hybrid functional B3LYP method with 6-311G(2d) basis set was used.

### 2.4. Computational Approach

#### 2.4.1. Molecular Modeling

TPPS structure was drawn and optimized using the Clean Geometry function of Discovery Studio Visualizer Software. The NH_4_ groups were removed from the structure for the molecular docking simulations, and we note the resulted structure TPPS.

The structures of our target proteins pro-caspase 3 (PDB code: 4JR0) [33] and caspase 3 (PDB code: 3DEI) [34] were imported from RCSB Protein Data Bank [35].

#### 2.4.2. ADME-Tox Predictions

ADME-Tox prediction were made using 3 web services pkCSM [36], admetSAR [37], and ProTox-II [38].

#### 2.4.3. Molecular Docking

We have predicted the interaction between TPPS and our target proteins: pro-caspase 3 and caspase 3 with AutoDock4.2.6 software [39] using our usual protocol [40,41]. We have performed blind docking simulations with the grid box grid points x y z (100, 110, 90) for human pro-caspase 3 respectively (126, 96, 82) for caspase 3. The spacing of the grid box is 0.375 Angstroms, and the Central Grid Point of Maps (x y z) is (6.46 1.11 23.11) for pro-caspase 3 and (−33.06 10.36 −9.75) for caspase 3.

### 2.5. In Vitro Bioassay

#### 2.5.1. Cell Culture

Human cutaneous melanoma cell line, Mel-Juso, (ACC-74, ATCC, Manassas, VA, USA) derived from vertical growth phase amelanotic melanoma was maintained in 75 cm^2^ culture flasks and RPMI (Roswell Park Memorial Institute, Buffalo, NY, USA) medium (1640, Gibco, Dublin, Ireland) supplemented with 1% antibiotics-antimycotics solution (Sigma-Aldrich, St. Louis, MO, USA) and fetal bovine serum (FBS, Gibco, by Life Technologies, Carlsbad, CA, USA) at a final concentration of 10%. At confluency of 80–90%, the cells were enzymatically detached from the culture flasks surface using a 0.25% trypsin–0.53 mM EDTA solution. Cell cultures were incubated in standard conditions (37 °C in an atmosphere with 5% CO_2_) and culture media was completely refreshed once every two days.

#### 2.5.2. Cell Treatment and LED Irradiation Protocol

To perform the in vitro experiments, Mel-Juso cells were seeded at a density of 5 × 104 cells/mL in 96 and 6-well plates and incubated at 37 °C, 5% CO_2_. After adhesion, the cells were treated with, γ-Fe_2_O_3_ NPs_TPPS, free γ-Fe_2_O_3_ NPs and free porphyrin, diluted in complete culture medium until a final concentration of 0.05, 0.5, 2.75, 4.16, 5.5 and 11 μg/mL for NPs, 0.01, 0.1, 0.5, 0.75, 1 and 2 μg/mL for porphyrin and 0.05 + 0.01, 0.5 + 0.1, 2.75 + 0.5, 4.16 + 0.75, 5.5 + 1 and 11 + 2 μg/mL for NPs + TPPS in γ-Fe_2_O_3_ NPs_TPPS, respectively. Untreated cells were used as control. To allow the internalization of porphyrin and NPs, cell cultures were incubated for 24 h at 37 °C, 5% CO_2_. Afterwards, the culture medium was replaced with a fresh one and the cells were irradiated or not for 0.5, 1, 2.5, and 5 min, and incubated at 37 °C. All experiments were performed at the interval of 24 h from the irradiation procedure. Before incubation with cells, the NPs and TPPS suspensions were sterilized using UVC radiation for 1 h. Verifications by absorption spectroscopy regarding possible photobleaching after UVC exposure were per-formed to be sure that the sterilization has no effect on suspensions. No changes of the spectra were noticed.

**Figure 2:**
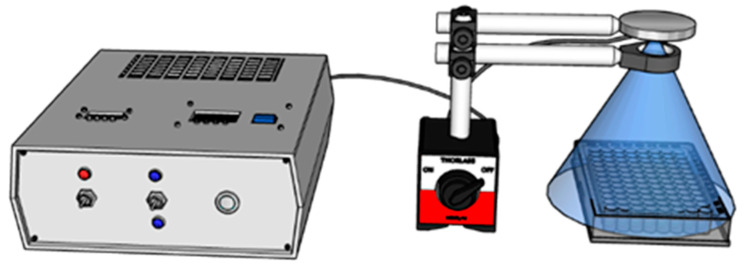
LED Irradiation experimental setup.

For cell exposure to UV light, a custom system of irradiation was developed (Figure 2). A 405 nm LED (Nikia, Japan, NVSU 233B) was used as a light source, with a radiant flux of 1400 mW. To control the intensity of LED and the time of irradiation, a Pulse Frequency Generator (PWM) and a Timer Relay Module were added to the pow-er supply circuit.

A Plano-Convex Lens, with the focal length of f = 100 mm, was placed between the LED and the plates, at a distance of 25 mm with respect to the light source to collect the entire radiation emitted by the diode (Figure 2), and 30 mm to the probe, to ir-radiate a large interest zone. The power density was measured at different duty cycles for the PWM, a calibration curve was drawn and used further in irradiation parameters selection.

#### 2.5.3. Cell Morphology Examination

After 24 h of LED irradiation, cell morphological changes were examined by phase-contrast microscopy using an Olympus IX73 microscope (Olympus, Tokyo, Japan) equipped with a Hamamatsu ORCA-03G camera (A3472-06, Hamamatsu, Japan). Representative images were acquired with the CellSens Dimension software (v1.11, Olympus, Tokyo, Japan).

#### 2.5.4. MTT Cell Viability Test

Melanoma cells were seeded at a density of 104 cells/well in 200 µL culture media and treated with porphyrin (0.01–2 μg/mL), NPs (0.05–11 μg/mL) and γ-Fe_2_O_3_ NPs_TPPS (0.05 + 0.01–11 + 2 μg/mL) for 24 h. After irradiation, Mel-Juso cells were incubated at 37 °C and the next day the culture media from each well was removed and replaced with 80 µL of 1 mg/mL MTT solution. Further, the culture plates were incubated for 2 h at 37 °C. In the final step, the purple formazan crystals formed during the incubation were solubilized with 150 µL isopropanol, and the absorbance of the samples was measured at 595 nm using a Flex Station 3 microplate reader (Molecular De-vices, San Jose, CA, USA). Non-irradiated cells were treated in the same conditions.

#### 2.5.5. Detection of Intracellular Reactive Oxygen Species (ROS) Production

Intracellular ROS production was measured using 2′,7′-dichlorodihydrofluorescein diacetate compound (H_2_DCFDA, D6883, Sigma-Aldrich, St. Louis, MO, USA). Mel-Juso cells were seeded in 96-well culture plates (5 × 10^4^ cells/mL) and treated with free TPPS (0.1–1 μg/mL), free NPs (0.5–5.5 μg/mL) and γ-Fe_2_O_3_ NPs_TPPS (0.5 + 0.1–5.5 + 1 μg/mL). After 24 h, the culture medium was removed and the cells were incubated for 30 min, at 37 °C with 50 μM H_2_DCFDA solution prepared in HBSS (Hanks’ Balanced Salt Solution). Further, H_2_DCFDA solution was removed, and the cells were irradiated or not for 1 min at a power density of 1 mW/cm^2^. The fluorescence was read at Flex Station 3 microplate reader (ex. 485 nm/em. 520 nm) after 24 h of incubation. The cells from each well were counted using the Trypan Blue method. The values of relative fluorescence units (RFU) were divided by cell number.

#### 2.5.6. Preparation of Cell Lysates

Mel-Juso cells were seeded in 6-well plates and treated with two different doses of TPPS (0.5 and 0.75 μg/mL) and the corresponding concentrations of NPs (2.75 and 4.16 μg/mL). After irradiation protocol and 24 h incubation, the cells were trypsinized. Further, the cell suspensions were centrifuged for 5 min, at 1500 rpm, 20 °C, and the cellular pellets were washed and resuspended in phosphate-buffered saline solution (PBS). The cell lysates were obtained by sonication of cell suspensions on ice, three times, using a Hielscher UP50H sonicator (80% amplitude, 1 cycle) and their centrifugation for 10 min, at 3000 rpm, 4 °C. The supernatants that resulted from centrifugation were collected and stored at −80 °C until biochemical determinations. The protein concentration of cellular lysates was estimated using Bradford reagent (B6916, Sigma-Aldrich, St. Louis, MO, USA) and a standard curve of 0–1.5 mg/mL bovine serum albumin (BSA).

#### 2.5.7. Measurement of Reduced Glutathione (GSH) Intracellular Content

The GSH amount of treated and untreated cells was determined using the method based on the reaction between GSH and Ellman’s reagent. Firstly, the cell lysates were deproteinized with an equal volume of 5% 5-sulfosalicylic acid and centrifuged for 10 min, at 10,000 rpm, 4 °C. Secondly, 10 μL of supernatant were mixed with 150 μL reaction mixture consisting of 1.5 mg/mL 5,5-dithio-bis-(2-nitrobenzoic acid) diluted in potassium phosphate buffer (K_2_HPO_4_/KH_2_PO_4_) 0.1 M, pH = 7, with 1 mM EDTA. Lastly, the samples were incubated at room temperature for 10 min and the absorbance was measured at 405 nm using a microplate reader. The GSH level of the samples was calculated using a 3.125–200 μM GSH standard curve.

#### 2.5.8. Western Blot Analysis

The expressions of some cell adhesion (β-catenin), proliferation (MCM-2), and apoptotic (caspase 3, Bax, NF-kB) markers were analyzed using the Western blot technique. The cell lysates were chemical (by mixing with a loading buffer containing SDS and β-mercaptoethanol) and thermic denatured (by heating at 95 °C for 5 min). Then, 40 μg of protein from each extract were loaded and migrated on denaturing 8%, 10%, and 15% SDS-polyacrylamide gels in TRIS-glycine-SDS buffer, for 2 h. Further, the proteins were electrotransferred from polyacrylamide gels on a PVDF membrane (cat. no. IPVH00010, Merck, Darmstadt, Germany) using a BioRad system and TRIS-glycine-methanol buffer. The proteins detection was performed using Western Breeze Chromogenic Anti-Mouse and Anti-Rabbit kits (WB7103, WB7105, Invitrogen, Carlsbad, CA, USA). The membranes were incubated with blocking solution for 30 min, at room temperature, and let overnight with monoclonal anti-MCM-2 (sc-373702, Santa Cruz, Biotechnology, Dallas, TX, USA), anti-β-catenin (sc-59737, Santa Cruz), and polyclonal anti-caspase 3 (sc-7148, Santa Cruz), anti-Bax (sc-493, Santa Cruz) and anti-NF-kB (sc-109, Santa Cruz) specific primary antibodies. The next day, the membranes were washed and then incubated for 30 min with conjugated-alkaline phosphatase secondary antibodies. The protein bands were revealed with BCIP/NBT chromogenic substrate, visualized with a ChemiDoc Imaging System (BioRad, Hercules, CA, USA) and quantified using ImageLab software. The results were normalized to a reference protein (β-actin).

#### 2.5.9. Statistical Analysis

The results were calculated as an average of three replicates (*n* = 3) and represent-ed as percentages relative to control (untreated cells) ± standard deviation. The statistical significance between treated cells and control was estimated using two-way ANOVA method performed with GraphPad Prism (Version 8, GraphPad Software, La Jolla, CA, USA) and Tukey’s multiple comparisons test and the results were considered significant when the *p*-value was less than 0.05 (*), 0.01 (**), 0.001 (***).

## 3. Results

### 3.1. Physicochemical Analysis

#### 3.1.1. Nanoparticle Characterization

**Figure 3:**
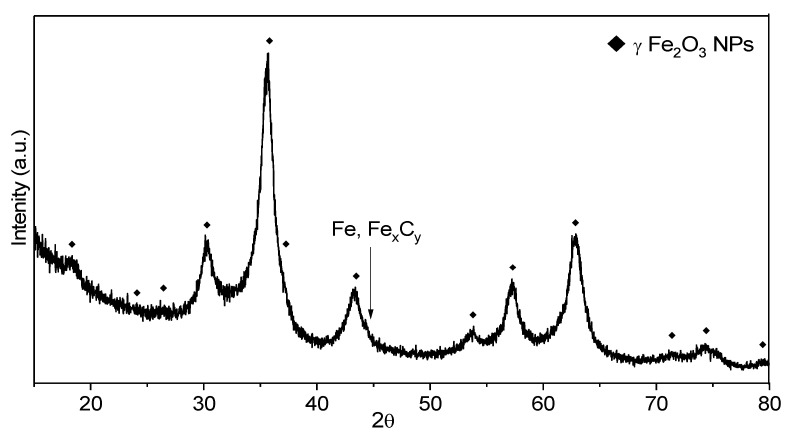
XRD patterns for as synthesized FeO1 nanoparticles (NPs).

The X-ray diffractogram (Figure 3) of the synthesized powder sample indicated its nanophase feature and the crystalline structure can be assigned to γ-Fe_2_O_3_/Fe_3_O_4_ phases: the position of theoretical diffraction peaks is illustrated with diamonds. The positions (2θ), and the full width at half maximum (B) of most relevant 5 peaks: (220), (311), (400), (511) and (440) were evaluated using pseudo-Voight functions and OriginPro 2015 software. The mean value for the constant lattice was 8360 Ǻ. The closest reference structure with that found for our sample is the γ-Fe_2_O_3_ phase having PDF file: 00-039-1346 and a constant lattice of 8352 Ǻ, whereas the magnetite (Fe_3_O_4_) phases have larger values (between 8.39 and 8.40 Ǻ).

Based on the most intense peak at 2θ = 35.6° associated with (311) plane and on the second one as the intensity at 2θ = 63.0° associated with (440) plane, the mean crystal size of as synthesized NPs was found to be 5.7 nm and 6.1 nm according to the Debye-Scherrer equation: Dhkl = k λ/B cosθ. Here, Dhkl is crystallite size, k is the shape constant of typical 0.9 in most evaluations for cvasi-spherical particles and λ is the wavelength of the X-ray. The presence of other iron-based crystalline phases (ex. αFe or Fe carbides) is not certain, their eventually minor contribution being masked by the background noise.

**Figure 4:**
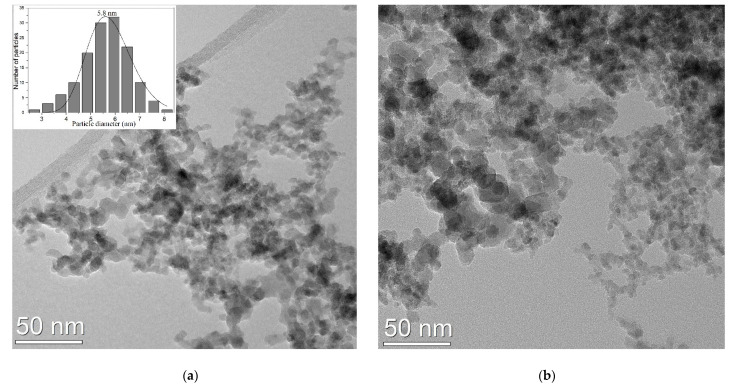
TEM images of the NPs (**a**) as synthesized nanoparticles (**b**) aggregates of NPs functionalised with TPPS.

The TEM image at medium resolution from the as synthesized NPs (Figure 4a) showed in majority cvasi-spherical structures which appear to have a diameter between 3–9 nm with a particles distribution well fitted with log-normal function and a mean value of 5.8 nm (left inset). The NPs, probably due to their magnetic properties, were self-organized in loose agglomerates/aggregates containing hundreds of entities. In the case of porphyrin functionalized NPs on copper grid, visualized by TEM in Figure 4b, the NPs agglomerates contained particles with almost the same dimensions, with a mean diameter of 6.2 nm, having more compact zones, yet with no evidence of solid segregation of porphyrin-based bridges between NPs or separate clusters. The most probable spreading of adsorbed porphyrins at the NPs surface almost as single molecular layer can be the explanation of this very weak mean diameter NPs increase.

**Figure 5:**
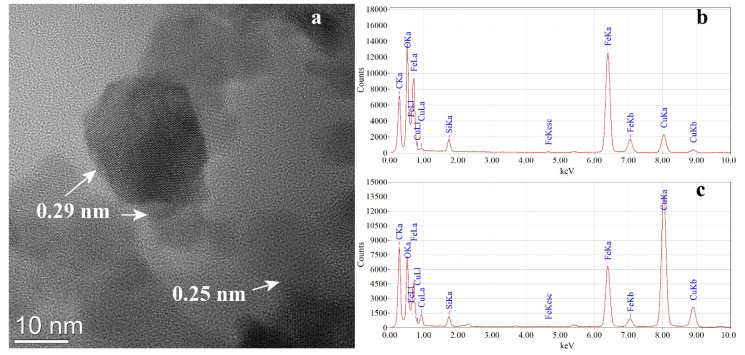
HR (high resolution) TEM Imageof NPs functionalized with TPPS (**a**) and EDS spectra for synthesized NPs (**b**), and NPs functionalized with TPPS (**c**).

As it can be seen from the presented HR-TEM image, (Figure 5a) the functionalized particles are spherical or polyhedral, the size of those identified here varying from 12 to 5 nm. The particles are mostly crystalline as monodomain and the observed interplanar distances can be ascribed to the (220) and (311) planes of γFe_2_O_3_/ Fe_3_O_4_ at 0.29 nm (2.96 Å) and 0.25 nm (2.52 Å) respectively. From both microscopes, NPs covered grids EDS analyzes were performed in different places where the NPs agglomerates are not attached to the original amorphous carbon layer of the TEM grids. Qualitatively the presence of O, Fe, and a minor contribution C was detected, all derived from NPs. The presence of S and the major contribution of C comes from the adsorbed pseudo-porphyrin whose molecules contain -SO_3_^−^ groups. The other elements appear as an artefact from the grids (Cu) or sample manipulation (Si). The constant presence of sulphur peak in EDS spectra from different places of the grid and also an intensity ratio between Fe-Kα and S-Kα peaks between 25 to 35 was used by us as an instrument to optimize the preparation process for NPs functionalized suspension.

A thin (less than 0.1 mm thick) pellet prepared by cold pressing from the as synthesized sample was analyzed by EDS as SEM facility in order to evaluate the elemental composition. Mediating the values from three different positions the following elemental composition was determined: C-0.63 at. %, O-58.60 at. %, and Fe-40.77 at.%. The Fe/O ratio from these values matches Fe_2_O_3–0.13_ stoichiometry.

Regarding the mean hydrodynamic size (Table 2), DLS measurements of the 0.5 g/L suspensions showed values of 138.0 nm for iron oxide nanoparticles and this value is consistent with the aggregate sizes measured at TEM investigation at low resolution. The higher value for the γγ-Fe_2_O_3_ NPs_TPPS suspension (453 nm) could be explained by coalescence and compactization phenomena of the initial NPs aggregates due to the strong centrifugated forces acting during the purification process; an effect that cannot be fully reversed by using the ultrasound redispersion.

**Figure 6:**
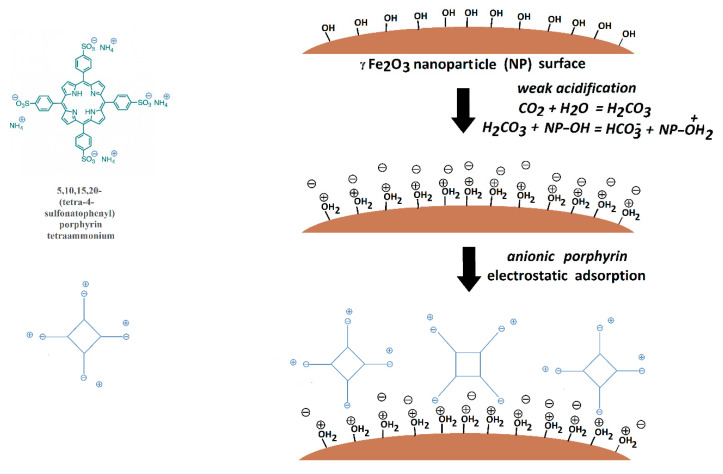
Schematic diagram of the adsorption process of the TPPS of the γ-Fe_2_O_3_ surface.

Zeta potential values (Table 3) indicated good stability for γ-Fe_2_O_3_ NPs (50.9 mV) and an excellent one for γ-Fe_2_O_3_ NPs_TPPS suspension (−90.9 mV) in slightly acidic dis-tilled water (due to dissolved CO_2_ from the atmosphere). This result can be explained by the electrostatic adsorption of the negatively charged surface porphyrin onto the iron oxide nanoparticles surface. A schematic diagram explaining the process was pro-posed in Figure 6.

#### 3.1.2. Spectroscopic Analysis

##### Absorption Spectroscopy

**Figure 7:**
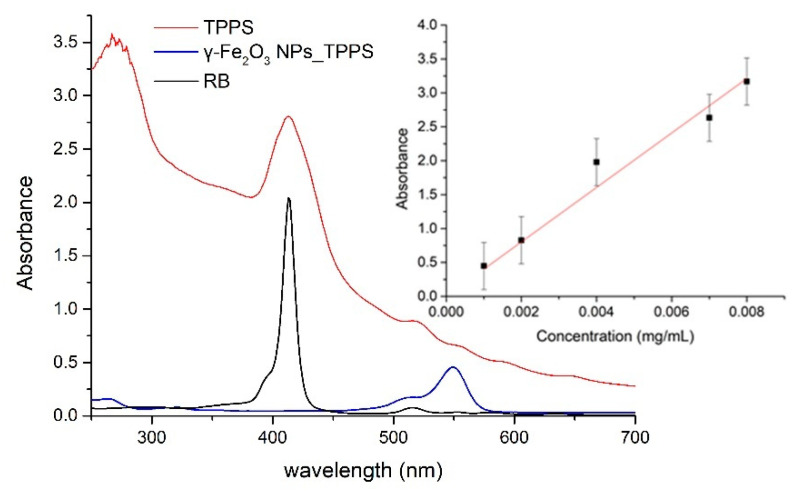
The absorption spectra for investigated samples: TPPS solution in distilled water at 0.004 mg/mL, γ-Fe_2_O_3_ NPs_TPPS water suspension, RB solution at 0.004 mg/mL in distilled water. In the inset, the standard curve of TPPS absorbance versus concentration is shown.

In Figure 7, the absorption spectrum for a TPPS solution in distilled water at concentration 0.004mg/mL is shown in comparison with the absorption spectrum for the water suspension of γ-Fe_2_O_3_ nanoparticles functionalized with TPPS (0.0138 mg/mL concentration of iron nanoparticles). The absorption features of porphyrin with maxima at 413 nm, 518 nm, 555 nm, and 649 nm are clearly present in the spectrum of nanoparticles suspension and proves the γ-Fe_2_O_3_ NPs and TPPS functionalization. In the inset of Figure 7, the standard curve for TPPS determined by the absorbance values for the peak at 413 nm at several concentrations is shown. Taking into account the absorbance of 1.04 for the γ-Fe_2_O_3_ NPs_TPPS suspension at 413 nm (background given by NPs diffusion subtracted), we can extrapolate from the standard curve a TPPS concentration of 0.0025 mg/mL in the γ-Fe_2_O_3_ NPs_TPPS complexes.

The samples were investigated by FTIR spectroscopy to highlight the functionalization of the nanoparticles with the TPPS.

**Figure 8:**
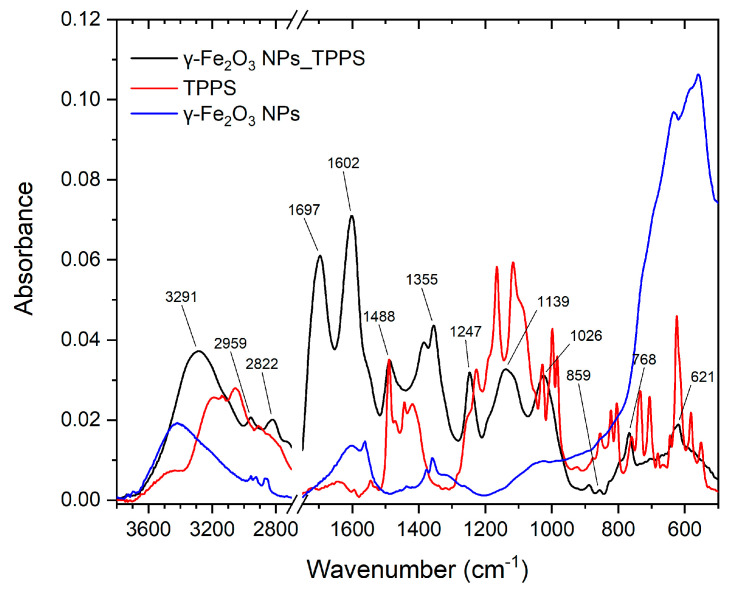
FT-IR spectra of TPPS, γ-Fe_2_O_3_ NPs, and γ-Fe_2_O_3_ NPs_TPPS.

Figure 8 displays the FT-IR spectra of TPPS, γ-Fe_2_O_3_ NPs, and complex γ-Fe_2_O_3_ NPs_TPPS.

The characteristics IR vibration of TPPS molecule were identified based on the existing literature [42,43,44,45] and the calculation molecular structure using Gaussian09 software. Table 4 presents the vibrational extracted from the experimental and theoretical spectra and their corresponding mode of vibration.

The IR spectra of TPPS presents the characteristic vibrations of the TPPS ring and the four SO_3_^−^ groups connected to the molecule phenyl moiety [42,43,44,45]. The N–H stretching vibration is identified at 3196 cm^−1^ and 3133 cm^−1^ and C–H stretching vibration is observed at 3056 cm^−1^ and 2907 cm^−1^. The bands at 1490 cm^−1^ and 1643 cm^−1^ are attributed to the stretching vibration of C−C and C=C bonds in the benzene ring corresponding to sulfonatophenyl radical and TPPS ring. The in-plane deformation vibration of C=C and C=N from the TPPS ring is observed at 1398 cm^−1^. The band at 1250 cm^−1^ represents the C−C stretching vibration between the aryl groups and the TPPS ring. The asymmetrical SO_3_ stretching vibrations are identified in the 1200–1100 cm^−1^ spectral range, with two intense bands at 1165 cm^−1^ and 1117 cm^−1^ [42]. The 1003 cm^−1^ and 738 cm^−1^ are assigned to N−H wagging vibration, whereas the band with peak at 624 cm^−1^ is attributed to the C−H wagging vibration of the benzene rings and deformation vibration of the TPPS ring.

The IR spectrum of NP shows a broad peak at 3417 cm^−1^ attributed to intermolecular hydrogen bond stretching vibration as the nanoparticle surface adsorbed the water. Further, this is supported by the appearance of the band with a peak at 1608 cm^−1^ representing the O–H bending vibration of water molecules adsorbed on iron nanoparticles [46,47,48]. The bands with peaks at 2957 cm^−1^, 2925 cm^−1^, and 2870 cm^−1^ correspond to the C–H stretching vibration, whereas the peaks at 1377 cm^−1^ and 1360 cm^−1^ are attributed to the C–H deformation vibration. In the range 770-450 cm^−1^ is observed the stretching mode characteristic to Fe-O bond vibrations, with a peak at 560 cm^−1^.

In the γ-Fe_2_O_3_ NPs_TPPS complex IR spectrum are identified the characteristic bands of TPPS at 1488 cm^−1^, 859 cm^−1^, 768 cm^−1^, 620 cm^−1^ and of γ-Fe_2_O_3_ are identified at 1601 cm^−1^, 1374 cm^−1^, and 1355 cm^−1^. The biggest changes are observed for the C–H and N–H vibrations of the TPPS ring. The bands at 1643 cm^−1^ and 734 cm^−1^ from the TPPS spectrum, related to stretching vibration of C−C/C=C and to N−H wagging vibration bonds from the TPPS ring, are shifted to 1697 cm^−1^ and 768 cm^−1^ in the γ-Fe_2_O_3_ NPs_TPPS complex IR spectrum. This indicates the interactions of TPPS with the surface of γ-Fe_2_O_3_ NPs that results in the stiffening of the TPPS ring [42,49] due to functionalization. More, a new band at 1247 cm^−1^ appears that indicates the functionalization of TPPS to γ-Fe_2_O_3_ due to the interaction of –OH of γ-Fe_2_O_3_ with -NH from the TPPS ring.

##### Singlet Oxygen Generation

**Figure 9:**
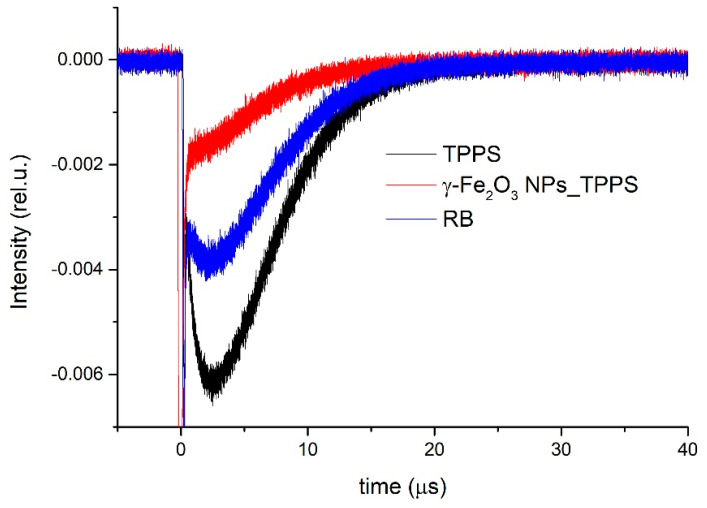
Time-resolved phosphorescence signals of singlet oxygen generated by the investigated samples.

Figure 9 shows the transient phosphorescence decay curves measured at 1270 nm for the singlet oxygen generated by the TPPS solution in distilled water, γ-Fe_2_O_3_ NPs_TPPS water suspension, and RB solution at 0.004 mg/mL in distilled water used as reference. The last has a known value of oxygen singlet quantum yield in water of 0.75 [50].

The phosphorescence signals were registered in the same experimental configuration and conditions for all investigated samples and averaged over 1000 laser shots. To avoid saturation effects, low energy of the laser excitation radiation of 3 mJ per pulse was used.

Using Φ_Δ_^*ref*^ value for RB in water of 0.75 [50] and Equation (1), we determined the quantum yield for singlet oxygen generation by γ-Fe_2_O_3_ NPs_TPPS complexes in water suspensions of 0.6. Also, the quantum yield of singlet oxygen generation by TPPS in water was found to be 0.8.

### 3.2. ADME-Tox Predictions

Since the compound is carried using the NP (so the Absorption and Distribution of the molecule may change), we focused on his toxicity. AdmeSAR and pkCSM predict that TPPS is soluble in water. TPPS has a 0.43 probability to be subcellular localized in the lysosome. Predictions show that this compound has a RatLD50 of 2.47 mol/kg according to admeSAR and 3066 mg/kg according to ProTox-II. This compound has a low toxicity class and probably is inactive as a carcinogen, immunotoxic, cytotoxic or mutagenic compound.

TPPS is inactive on the AhR receptor, the activation of this receptor being linked to immunosuppression, thymic involution, and immunotoxicity via transcriptional alterations [51]. The decrease of the mitochondrial membrane potential is associated with apoptosis [52], the prediction of inactivity of the TPPS derivative on mitochondrial membrane potential could suggest that this compound may not produce apoptosis.

TPPS is also inactive on p53 and ATAD5. Regarding hepatotoxicity, pkCSM and ProTox-II web services reported that this compound is not hepatotoxic. Overall, according to predictions made, TPPS is a non-toxic compound. We present the predicted results in Table 5.

### 3.3. Molecular Docking Simulations

Molecules with estimated binding energy greater than −6 kcal/mol have no biological action on that target [53,54]. When TPPS interacts with Pro-caspase3, our results show that it has the lowest predicted binding energy −9.31 kcal/mol (Table 6).

**Figure 10:**
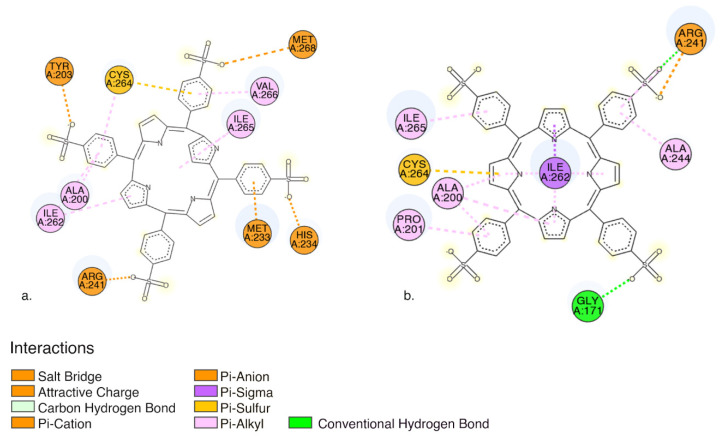
2D visualization of (**a**) interactions between TPPS and pro-caspase3 amino acid residues (**b**) interactions between TPPS and caspase3 amino acid residues. The image was obtained using Discovery studio visualizer. (Dassault Systèmes (V20.1.0.19295), San Diego, CA, USA).

Pro-caspase 3 has the lowest predicted binding energy compared to caspase 3, implying that TPPS will most likely bind to pro-caspase (Table 6). In both cases, between TPPS and caspases, we obtained favorable interactions as follows: (i) non-classical carbon-hydrogen bound, (ii) electrostatic interactions, (iii) charge interactions: salt bridge, and attractive charge; (iv) Pi-charge: Pi-cation and Pi anion; (v) hydrophobic: Pi- Alkyl, Pi-Sigma interactions; and (iv) miscellaneous Pi-sulphur interactions (Figure 10).

The interaction situs in both cases is quite similar both pro-caspase 3 and caspase 3 form favourable interactions with amino acid residues: Ala200, Arg241, Ile 262, Cys264, Ile 265 (Figure 10).

### 3.4. In Vitro Biological Studies

#### 3.4.1. Phototoxicity and Cell Morphologic Alterations

**Figure 11:**
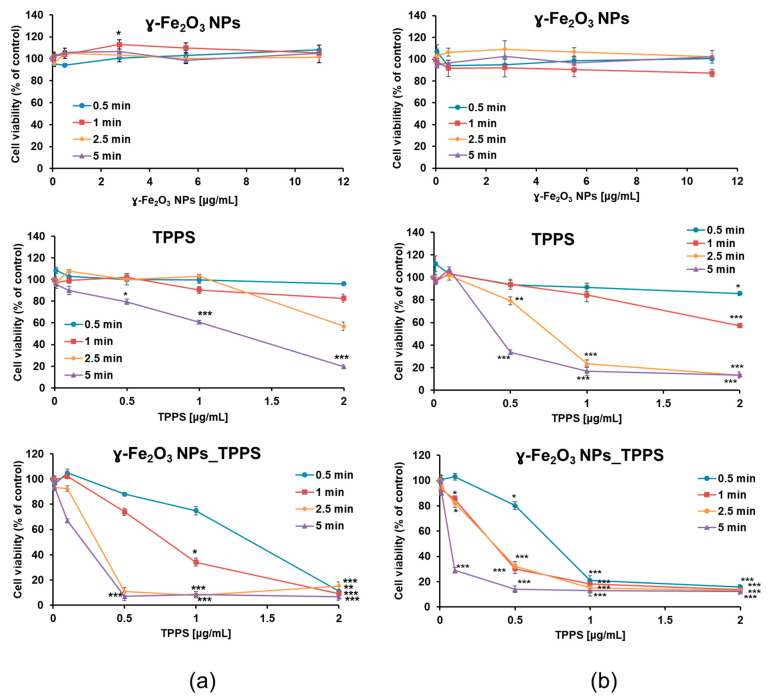
Variation of Mell-Juso cell viability depending on irradiation time (min), led power density (mW/cm^2^) and treatment dose. Cells were incubated for 24 h with γ-Fe_2_O_3_ NPs, TPPS and their complex at different concentrations and then irradiated for 0.5, 1, 2.5 and 5 min at (**a**) 1 and respectively (**b**) 2 mW/cm^2^ power densities. After 24 h from irradiation cell viability was evaluated by MTT assay. Data (*n* = 3) were expressed as percentages related to control (100%) ± standard deviation (SD). The results were considered statistically significant when * *p* < 0.05; ** *p* < 0.01; *** *p* < 0.001 (sample vs. control).

The photodynamic activity of γ-Fe_2_O_3_ NPs_TPPS complexes was evaluated in comparison with that of the individual components of the system and performed by MTT assay. After 24 h after treatment, the Mel-Juso cells were washed and then irradiated at 405 nm at 2 different power densities of 1 mW/cm^2^ (Figure 11) and respectively, 2 mW/cm^2^ (Figure 11b) for different time intervals (0.5, 1, 2.5 and 5 min). After irradiation, cells were incubated for another 24 h. As demonstrated in Figure 11, the phototoxicity of TPPS and γ-Fe_2_O_3_ NPs_TPPS on melanoma cells increased with the elevation of led power density and irradiation time. Moreover, the suppression of cell viability was dependent on the TPPS concentration. For example, the viability of cells ex-posed to 1 μg/mL TPPS decreased by 40% compared to control after 5 min irradiation with 1 mW/cm^2^ power density and by 80% when exposed to 2 μg/mL in the same conditions. The results revealed that none of the applied conditions had a significant effect on cells treated with free γ-Fe_2_O_3_ NPs, except for a slight increase corresponding to the dose of 2.75 μg/mL, thus demonstrating their biocompatibility. However, the phototoxicity of TPPS was significantly enhanced in the presence of NPs. A pronounced reduction of cell viability was observed starting with a dose of 0.5 μg/mL TPPS in cells incubated with γ-Fe_2_O_3_ NPs_TPPS compared with the one with free TPPS. As shown in Figure 11b, at 2 mW/cm^2^ power density, regardless the irradiation time, cells that were treated with a concentration higher than 1 μg/mL TPPS presented a very significant decrease of cellular viability. In comparison, at 1 mW/cm^2^ power density, a higher per-centage of cells survived only when irradiated for 0.5 and 1 min. Based on these results, irradiation of cells at 1 mW/cm^2^ power density for 1 min was considered the most suitable setup for the next determinations.

**Figure 12:**
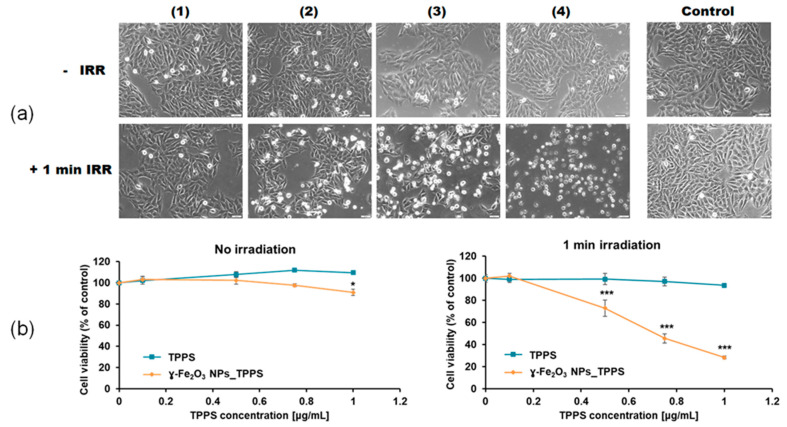
The effect of no irradiation vs. irradiation (λ ~ 405 nm, 1 mW/cm^2^, 1 min) conditions on morphology and viability of treated Mel-Juso cells. (**a**) Bright-field images of Mel-Juso cells after 24 h exposure to different concentration of γ-Fe_2_O_3_ NPs_TPPS complex: (1) 0.5 μg/mL NPs and 0.1 μg/mL TPPS; (2) 2.75 μg/mL NPs and 0.5 μg/mL TPPS; (3) 4.16 μg/mL NPs and 0.75 μg/mL TPPS; (4) 5.5 μg/mL NPs and 1 μg/mL TPPS and subjected or not to led irradiation. Magnification 10X. Scale bar is 50 μm. (**b**) Cell viability of Mel-Juso cells after 24 h incubation with TPPS and γ-Fe_2_O_3_ NPs_TPPS complex at different concentrations. Data (*n* = 3) were expressed as percentages related to control ± standard deviation (SD). The results were considered statistically significant when * *p* < 0.05; *** *p* < 0.001 (sample vs. control).

Using these selected parameters, we evaluated the morphologic aspect of Mel-Juso cells and their viability after incubation with γ-Fe_2_O_3_ NPs_TPPS complexes and LED irradiation (Figure 12a,b). The morphologic aspect of non-irradiated cells was similar to that of the control, which is characteristic for epithelial cells presenting smooth, unruffled borders. When cells were irradiated for 1 min, the cells changed their aspect dependent by TPPS concentration. A significant decrease of cell density was noticed after incubation of cells with γ-Fe_2_O_3_ NPs_TPPS complexes starting from a dose of 0.5 μg/mL TPPS. As observed in Figure 12a, plenty of cells detached from the substrate acquiring a round shape with irregular borders. Non-specific cell extensions and apoptotic vesicles were also detected at a dose higher than 0.75 μg/mL TPPS.

The alterations noticed in the microscopic images were confirmed by MTT assay (Figure 12b). Without irradiation, exposure of Mel-Juso cells to γ-Fe_2_O_3_ NPs_TPPS complexes or free TPPS generated no significant toxicity except for the dose of 1 μg/mL γ-Fe_2_O_3_ NPs_TPPS complexes where viability slightly decrease by 10%. When irradiated, melanoma cells incubated with γ-Fe_2_O_3_ NPs_TPPS complexes presented a 45% viability compared to control at a dose of 0.75 μg/mL TPPS. However, no significant changes were observed for cells incubated with free TPPS.

#### 3.4.2. ROS Production and Antioxidant Protection

**Figure 13:**
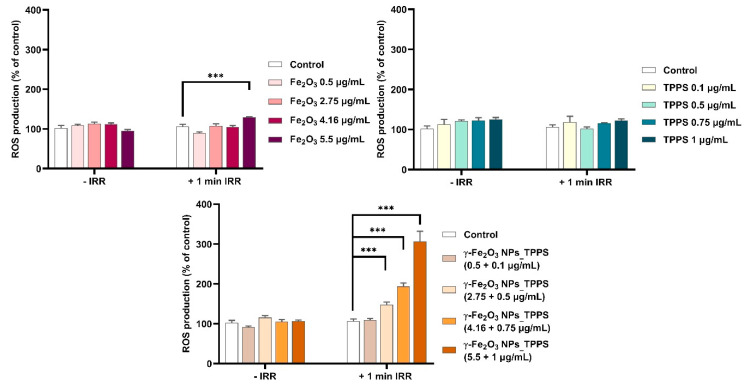
Intracellular ROS production after incubation for 24 h with different concentrations of γ-Fe_2_O_3_ NPs, TPPS and their complex and exposure to no irradiation vs. 1 min irradiation (λ ~ 405 nm, 1 mW/cm^2^) conditions. Data (*n* = 3) were expressed as percentages related to control ± standard deviation (SD). The results were considered statistically significant when *** *p* < 0.001 (sample vs. control).

PDT-induced cell death generally occurs through the generation of intracellular ROS. Therefore, we measured intracellular ROS levels in melanoma cells to evaluate the photodynamic activity of synthesized complexes. As shown in Figure 13, under irradiation, the ROS production increased significantly in a dose-dependent manner in cells treated with γ-Fe_2_O_3_ NPs_TPPS complexes starting with a dose of 2.75 μg/mL NPs and 0.5 μg/mL TPPS respectively. Interestingly, no change of ROS level was detected in cells treated with free TPPS or γ-Fe_2_O_3_ NPs excepting the highest dose of NPs where a slight elevation of ROS production was found. In non-irradiated conditions, the level of ROS in treated cells was similar to that of control.

**Figure 14:**
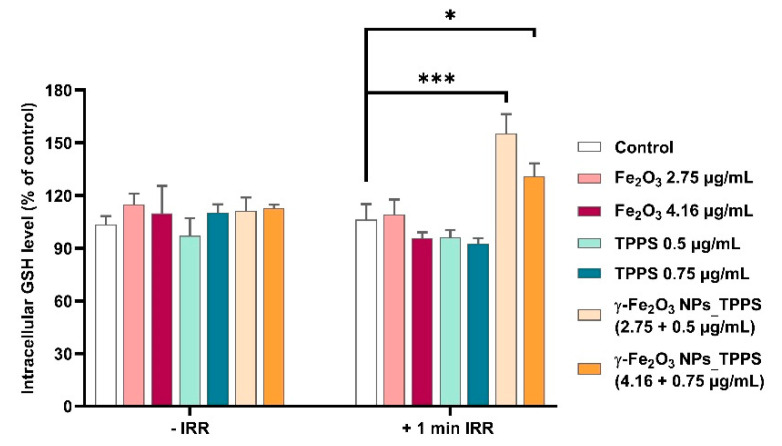
Variation of reduced glutathione (GSH) content in Mel-Juso cells incubated for 24 h with 2 different doses of γ-Fe_2_O_3_ NPs, TPPS and their complex and submitted or not to 1 min led irradiation (λ ~ 405 nm, 1 mW/cm^2^). Data (*n* = 3) were expressed as percentages related to control ± standard deviation (SD). The results were considered statistically significant when * *p* < 0.05; *** *p* < 0.001 (sample vs. control).

In order to evaluate the antioxidant defense response of treated cells exposed or not to irradiation, the intracellular GSH level was measured (Figure 14). When irradiation was applied for 1 min, the GSH level increased significantly by 55% and 31% respectively compared to control in cells treated with the two selected doses of γ-Fe_2_O_3_ NPs_TPPS. In cells that were not exposed to LED light, we found that the GSH content of treated cells was almost similar to the one measured in control cells.

#### 3.4.3. Protein Expression of Proliferation, Cell Adhesion and Apoptosis-Related Markers

**Figure 15:**
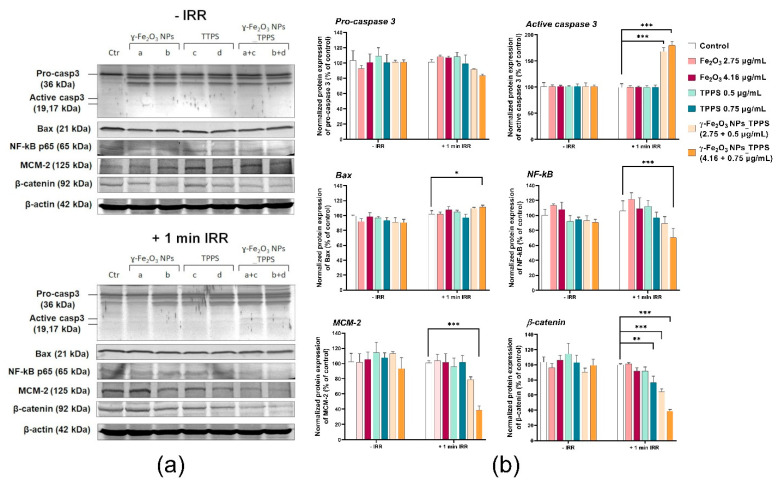
The protein expression of Pro-caspase 3, caspase 3, Bax, NF-kB, MCM-2 and β-catenin in Mel-Juso cells after 24 h incubation with 2 different concentrations of γ-Fe_2_O_3_ NPs, TPPS and their mixture and exposure to no irradiation vs. 1 min irradiation (λ ~ 405 nm, 1 mW/cm^2^) conditions. (**a**) Blot bands corresponding to control (Ctr), γ-Fe_2_O_3_ NPs (a. 2.75; b. 4.16 μg/mL), TPPS (c. 0.5; d. 0.75 μg/mL) and γ-Fe_2_O_3_ NPs_TPPS complex (a+c, b+d); (**b**) Quantification of blot images. Data (*n* = 3) were normalized to β-actin and expressed as percentages related to control (100%) ± standard deviation (SD). The results were considered statistically significant when * *p* < 0.05; ** *p* < 0.01; *** *p* < 0.001 (sample vs. control).

To demonstrate the anti-tumor potential of γ-Fe_2_O_3_ NPs_TPPS in combination with LED irradiation, Western blotting analysis was performed on three apoptosis-related proteins, caspase 3, Bax and NF-kB, a proliferation marker MCM-2 and a com-ponent of adherens junctions, that promotes cell adhesion, β-catenin (Figure 15). The results showed a significant decrease of MCM-2 and β-catenin protein levels dependent on TPPS concentration in the Mel-Juso cells treated with γ-Fe_2_O_3_ NPs_TPPS and irradiated for 1 min at 1 mW/cm^2^ power density. A slight decrease was also noticed in cells treated with free TPPS in a dose of 0.75 μg/mL. No significant changes in the ex-pression of these two proteins were observed in any of the other conditions. The protein level of pro-caspase 3 decreased concomitantly with the increase of caspase 3 ex-pression, resulting in apoptosis activation in Mel-Juso cells treated with γ-Fe_2_O_3_ NPs_TPPS complexes and exposed to irradiation. Elevation of Bax expression, a pro-apoptotic protein, was observed only in cells treated with the higher dose of γ-Fe_2_O_3_ NPs_TPPS (0.75 μg/mL TPPS). For the same condition, we observed an inhibition of NF-kB protein expression compared to control cells, which confirm induction of apoptosis and suppression of proliferation. For non-irradiated cells, using the same doses and exposure intervals, no significant changes in the expression of the analyzed proteins were found.

## 4. Discussion

To evaluate the potential of the newly synthesized γ-Fe_2_O_3_ NPs_TPPS complexes in melanoma PDT, their anti-tumoral activity was investigated on amelanotic Mel-Juso cells exposed to 405 nm LED irradiation. The blue light treatment at 450 nm wavelength and 10 J/cm^2^ had better results than red light in an in vitro melanoma study [55]. The efficiency and the safety of red (635 nm) and blue (400 nm) light in PDT of basal cell carcinoma seems to be equally safe and effective [56], but porphyrins expect to deliver better results at 410 nm than in red light [57]. In melanoma PDT treatment, flavin mononucleotide shows good results after blue light irradiation (450 nm), resulting in an 85–90% inhibition of tumour growth [58]. Although the low depth penetration of blue light (at 400 nm of 1 mm [59]), the low expression of melanin pigment present in Mel-Juso cells [60,61] can suggest that the 405 nm LED light only induces the activation of porphyrin derivate, without its absorption by melanin. Our results indicated the efficiency of the PDT procedure at 405 nm in vitro on Mel-Juso melanoma cells. Although, the low skin penetration depth that characterizes blue light limits the PDT impact in vivo, it can be applied to superficial tumors. The blue light was already used in clinical trials to treat actinic keratoses [62,63] and it was suggested that blue light improved skin texture and smoothness and that can be used for treating photodamaged skin. The blue light is potentially safe and effective and could be employed for treatment of acne, acne rosacea, and sun-damaged skin [62]. Also, in [58] has been reported a melanoma xenograft regression in mice (85–90%) after blue light photoactivation. Taking into account all of these, future research is worth to be carried out for increasing the outcome of blue light PDT by controlling the PS activation and the reactions in the tegument tissue layers.

Previous studies showed that synthetic anionic porphyrins present high photoactivity and, accumulate rapidly and preferentially in tumor cells [64,65]. However, their use has been associated also with several disadvantages including aggregation, dark toxicity (in the absence of light) and side effects (e.g., neurotoxicity) which has impeded its development as a PS [65]. Using iron oxide nanoparticles as a vehicle can solve these disadvantages. Apart from being a PS agent, TPPS also improved the stability of the γ-Fe_2_O_3_ NPs_TPPS suspension and its aggregation significantly decreased. Moreover, using γ-Fe_2_O_3_ in combination with PS can significantly enhance the PDT efficiency, apart from eliminating the side effects of simple PS. Also, due to the magnetic properties of these vehicles they can be used as a targeted method, thus increasing the selectivity of the PDT [11]. The strategy to combine porphyrin with NPs may lead to an increase of the delivery of this compound within the target cells, thus leading to the enhancement of the porphyrin photoactivity in PDT at smaller doses and to the reduction of systemic toxicity.

Our results showed that γ-Fe_2_O_3_ NPs_TPPS has an increased phototoxicity compared with free TPPS. The preliminary cytotoxicity studies revealed that the phototoxicity of γ-Fe_2_O_3_ NPs_TPPS is highly dependent on irradiation time, LED power density, and TPPS concentration. The Mel-Juso cell viability decreased dramatically with over 80% from control upon irradiation with 1 and 2 mW/cm^2^ power densities for 5 min in the presence of γ-Fe_2_O_3_ NPs_TPPS corresponding to a TPPS concentration ≥0.5 μg/mL. A different response was obtained in cells treated with free TPPS, in the same conditions. Here, the phototoxicity was significantly lower compared to the one of functionalized TPPS. No significant alteration of cell viability was noticed in cells incubated with free NPs which attests their high biocompatibility but also might suggest their capability of no interference in PDT. Considering these results, optimal experimental conditions for further analysis were established at 1 min irradiation and 1 mW/cm^2^ power density.

Furthermore, we found that in the absence of irradiation, the melanoma cell viability has not been altered by incubation with TPPS or γ-Fe_2_O_3_ NPs_TPPS up to a concentration of 1 μg/mL TPPS. Also, we showed that unfunctionalized TPPS did not exert toxicity under irradiation up to a concentration of 1 μg/mL. These results might indicate a threshold of TPPS cytotoxicity in the melanoma cells subjected or not to irradiation at 1 μg/mL. A dose of 0.75 μg/mL of functionalized TPPS was necessary to reduce approximately half of the melanoma cell population (IC50 dose) after 1 min irradiation followed by 24 h of incubation. A similar study showed that a 50% lethal concentration for TPPS applied on human skin melanoma G361 cells was 4.24 ± 0.12 μM, after 50 s of irradiation with a dose of 1 J/cm^2^ and 24 h incubation of cells [66]. After a microscopic examination, we noticed significant morphologic alterations of melanoma cells incubated with γ-Fe_2_O_3_ NPs_TPPS which correspond to dead cells including unspecific cell extensions, ruffle borders shrinkage, blebbing or apoptotic vesicles. Many detached cells floating in the culture media were also present.

Despite the fact that TPPS is a good photosensitizer with a quantum yield of singlet oxygen generation of about 80% (determined via phosphorescence detection at 1270 nm), at concentrations up to 1 μg/mL and at a very low dose of light irradiation (1 mW/cm^2^ and 1 min exposure), it did not show a PDT effect, in contrast with the iron oxide nanoparticles having the same TPPS loading. Our studies showed that γ-Fe_2_O_3_ NPs has led to a significant increase of TPPS photodynamic activity.

The photophysical studies showed for γ-Fe_2_O_3_ NPs_TPPS, a quantum yield of singlet oxygen generation of 60%, slightly lower than the porphyrin alone. These suggest that the significant ROS increase is due to the ability of synthesized NPs to deliver the photosensitizer intracellularly.

The photooxidation efficiency of TPPS in melanoma cells increased in a concentration dependent manner when it was added in combination with NPs. These results are in correlation with the above cytotoxicity results and might suggest the contribution of NPs in the TPPS cell internalization and its accumulation in the cytosol. Our assumption is based on previous studies regarding the internalization of iron oxide NPs. It was shown that endocytosis is the most frequent mechanism of cellular uptake for this kind of nanoparticles [67]. More intracellular uptake pathways of iron oxide NPs were proposed including passive diffusion, micropinocytosis, receptor-mediated endocytosis, clathrin-mediated endocytosis, caveolin-mediated internalization, and other clathrin and caveolin-independent endocytosis [68]. Furthermore, several research studies indicated that the incubation with a higher concentration of NPs results in a higher internalization that could reach a maximum after 24 h of exposure [69]. In melanoma cells, it was shown that internalization of superparamagnetic iron oxide nanoparticles coated with polyvinyl alcohol and functionalized with amino groups is visible after 24 h of continuous exposure [70]. In another study, on human melanoma cells, it was shown that exposure to ultrasmall superparamagnetic iron oxide nanoparticles induced a clathrin-mediated uptake with the involvement of the endosomal-lysosomal pathway [71].

Iron oxide NPs are internalized into cells usually by endocytosis and digested to iron ions inside lysosomes where also NP-functionalized compounds are released in cytosol. This localization might contribute to the increase of efficiency of γ-Fe_2_O_3_ NPs_TPPS on cancer cells. The PDT efficacy might be also partly due to a release of lysosomal constituents such as cathepsins to the cytosol along with TPPS [72]. The released iron ions could also enhance PDT efficacy by contributing to the increase of ROS production. Iron ions are involved in catalyzation of the Fenton reaction, which produces highly reactive hydroxyl radical (OH•) from peroxide (H_2_O_2_) and superoxide anions (O_2_^−^).

In cells, ROS production is balanced by enzymatic and non-enzymatic antioxidant defense systems. An excess of ROS induces the loss of antioxidant capacity and macromolecular damage thus triggering oxidative stress. In this direction, we further evaluated the intracellular GSH level in melanoma cells. This most abundant endogenous thiol compound, is a non-enzymatic antioxidant with important roles in maintaining redox homeostasis through direct scavenging or enzymatic catalysis of electrophilic and oxidant species [73,74]. The elevated GSH level found in tumor cells confers them therapeutic resistance by preserving the levels of cysteine and detoxification of xenobiotics [75]. Previous studies suggest that overexpression of GSH might be also correlated with resistance to PDT [76,77]. In the current study, we found that the treatment with γ-Fe_2_O_3_ NPs_TPPS under 1 min irradiation induced a slight elevation of GSH synthesis compared to control as a response to high ROS production. Moreover, we noticed that the increase of GSH content was inversely proportional with the increase of ROS level. These data indicate that the cells were not able to efficiently counteract the oxidative stress. Once installed, the oxidative stress can lead to significant oxidative modifications, including protein oxidation, lipid peroxidation, DNA damage, inflammation, perturbation of signaling pathways and activation of cell death mechanisms [78].

To further investigate the anti-tumor mechanisms triggered in melanoma cells following photodynamic activation of functionalized TPPS, we analyzed some specific protein markers involved in different cellular processes such as apoptosis, proliferation and cell adhesion. We obtained a decrease of pro-caspase 3 protein level in parallel with the activation of caspase 3 in cells incubated with γ-Fe_2_O_3_ NPs_TPPS at 0.5 μg/mL and 0.75 μg/mL TPPS and irradiated for 1 min. These results indicate activation of apoptosis in melanoma cells as cell death mechanisms. Additionally, in the same cells, we found a slight upregulation of Bax protein, a major member of the pro-apoptotic group of the Bcl-2 family, which can activate caspase-3 through the intrinsic apoptotic pathway. Because of the small increase of Bax protein expression, we assume that activation of caspase 3 in Mel-Juso cells was achieved possibly by multiple pathways. Inhibition of NF-kB (nuclear factor kappa-light-chain-enhancer of activated B cells) in these cells also confirmed the induction of apoptosis but also the suppression of proliferation [79]. NF-kB plays a critical role in the cellular response to different stimuli including stress, free radicals and ultraviolet irradiation and controls signaling pathways involved in cell survival. The TPPS-mediated apoptosis was reported also in human gastric cancer HGC27 and SNU-1 cells treated with a dose of 6.25 µM TPPS derivate and irradiation by a laser at 650 nm with 12 J/cm^2^ [80].

In cells, DNA replication process is assisted by minichromosome maintenance proteins (MCMs), considered also proliferation markers and key tools in the diagnosis and prognosis of cancer [81]. Very recently, it was found that melanoma is characterized by elevated expressions of MCM-2–6 and MCM-10 proteins [82]. Moreover, MCM-2-index is correlated with lower survival rates and this protein in association with COX-2 and p-Akt1 contribute to cell-cycle dysregulation in melanoma [83]. Considering this, we have analyzed the expression of MCM-2 protein to evaluate the status of the proliferative capacity of tumor cells under tested conditions. The bands revealed on blot membranes showed a decreased expression of MCM-2 protein in Mel-Juso cells exposed to γ-Fe_2_O_3_ NPs_TPPS followed by irradiation, which confirmed the inhibition of melanoma cells proliferation. These results were also in correlation with the cytotoxicity evaluation and morphologic examination data. We assume that the decrease of MCM-2 protein expression may be a consequence of ROS overproduction induced in melanoma cells through TPPS activation after 1 min irradiation in the presence of γ-Fe_2_O_3_ NPs_TPPS. It is well known that overproduction of ROS is positively correlated with cell cycle arrest and apoptosis. ROS are highly active oxygen-containing molecules that play an essential role in cell cycle progression by regulating the ubiquitination process via intermediate phosphorylation of cyclins-dependent kinases (CDKs) and cell cycle regulatory molecules. MCM-2 is one of the molecules that regulate cell proliferation and the cell cycle via G1/S phase arrest. The regulatory mechanisms that modulate and control its activity are diverse and complex, particularly, phosphorylation by multiple kinases [84]. Compelling evidence showed that the decrease of MCM-2 expression may be a consequence of downregulation of CK2α the catalytic subunits of protein kinase CK2 [85], or a result of Notch-mediated cell cycle arrest and its dependent kinases [86].

Furthermore, the protein expression of β-catenin, a structural component of cell–cell adhesions was also investigated. The role of β-catenin in melanoma is not completely understood. It’s up-regulation has been linked to the suppression of melanoma cell invasion [87], but also was reported that blockade of β-catenin in metastatic melanoma cell lines induces apoptosis and inhibits proliferation, migration and invasion but not in primary melanoma cell lines [88]. In the current study, we found that protein expression of β-catenin was pronouncedly down-regulated in our primary melanoma cells when incubated with γ-Fe_2_O_3_ NPs_TPPS and then irradiated. A slight decrease in its expression was also registered in the irradiated cells treated with free TPPS at the highest concentration. The reduction of β-catenin protein expression may result following ROS generation. Previously, it was shown that the degradation of β-catenin in epidermal cells is caused by ROS accumulation through caspases activation. Simultaneously, damage of cell adhesion may induce ROS production and caspase activation, indicating a loop mechanism involved in cell death [89].

Taking all results into consideration, we tend to believe that in this case, suppression of β-catenin contributes to the induction of apoptosis, suppression of proliferation and reduction of cell adhesion.

## 5. Conclusions

This study proved the anti-tumoral effect of the newly synthesized γ-Fe_2_O_3_ NPs functionalized with 5,10,15,20-(Tetra-4-sulfonatophenyl) porphyrin on human melanoma cells subjected to PDT by 405 nm LED irradiation. The NPs were synthesized by laser pyrolysis and their nanometric size and crystallinity were confirmed by DLS, XRD and TEM analyses. The loading efficiency of NPs with TPPS was estimated by using absorption spectroscopy.

The γ-Fe_2_O_3_ NPs_TPPS complexes showed a good efficiency for singlet oxygen generation, a quantum yield of 60% being determined by measurements of ^1^O_2_ phosphorescence at 1270 nm.

Predicted toxicity results suggested that this compound is probably safe to use, but future tests are needed. The low binding energy between TPPS and pro-caspase 3 suggests a possible affinity of TPPS to the inactive form of caspase.

The biological investigations showed that γ-Fe_2_O_3_ NPs has led to a significant increase of TPPS photodynamic activity for a very low irradiation dose (1 mW/cm^2^ irradiation intensity and 1 minute of exposure). Despite the fact that TPPS is a good photosensitizer with a high quantum yield of singlet oxygen generation, it did not show a PDT effect at our low light dose, in contrast to iron nanoparticles loaded with the same concentration of TPPS. This could possibly indicate a good cell internalization rate of newly synthesized γ-Fe_2_O_3_ NPs functionalized with TPPS.

A threshold of TPPS cytotoxicity in melanoma cells exposed or not to blue light radiation was found at 1 μg/mL. Moreover, we showed that photoactivated γ-Fe_2_O_3_ NPs_TPPS could trigger apoptosis in melanoma cells and might suppress tumor proliferation and cell adhesion by modulating the MCM-2 and β-catenin pathways.

## Figures and Tables

**Table 1:** Experimental data.

Sample	D-C_2_H_4_/Fe(CO)_5_	D-synth. Air	D-Ar Conf	D-Ar_windows_	T_flame_	P	P_laser_
unit	sccm	sccm	sccm	sccm	°C	mbar	W
FeO1	33/6.6	33	750	250	670	300	157/150

**Table 2:** The mean hydrodynamic size of the samples.

Sample	PI	Z-Average	Distribution Form
γ-Fe_2_O_3_ NPs	0.340	138.0 nm	Broad polydisperse
γ-Fe_2_O_3_ NPs _TPPS	0.414	453.1 nm	

**Table 3:** Zeta potential values for the γ-Fe_2_O_3_ NPs and γ-Fe_2_O_3_ NPs_TPPS samples

Sample	γ-Fe_2_O_3_ NPs	γ-Fe_2_O_3_ NPs_TPPS
Zeta Potential (Mean)	50.9 mV	−90.9 mV
Electrophoretic Mobility Mean	0.000394 cm^2^/Vs	−0.000704 cm^2^/Vs

**Table 4:** Experimental and theoretical vibration frequencies for TPPS with their corresponding modes of vibrations.

Wavenumber (cm^−1^)		Mode of Vibration
Experimental	Theoretical	
1643	1620	stretching vibration of C−C and C=C bond (sulfonatophenyl radical)
1544	1559	stretching vibration of C−C and C=C bond (porphyrin ring/sulfonatophenyl radical)
1490	1477	stretching vibration of C−C and C=C bond (porphyrin ring/sulfonatophenyl radical)
1443	1436	stretching vibration of C−C and C=C bond (sulfonatophenyl radical)
1418	1416	stretching vibration of C−C and C=C bond (porphyrin ring)
1398	1346	in-plane deformation vibration of C=C and C=N (porphyrin ring)
1250	1252	C−C stretching vibration
1226	1213	C−H deformation vibration
1165	1154	symmetrical stretching vibrations S−O
1117	1124	asymmetrical stretching vibrations S−O
1025	1036	N−H and C−H deformation vibrations
998	1003	N−H wagging vibration
984	985	N−H and C−H deformation vibrations (porphyrin ring)/deformation vibrations of 4 neighbouring H-atoms (sulfonatophenyl radical)
855	839	deformation vibrations of 4 neighbouring H-atoms (sulfonatophenyl radical)
805	804	deformation vibrations of 4 neighbouring H-atoms (sulfonatophenyl radical) C−C skeletal vibration (porphyrin ring)
760	767	deformation vibrations of 4 neighbouring H-atoms (sulfonatophenyl radical)
734	740	deformation vibrations of 4 neighbouring H-atoms (sulfonatophenyl radical)
681	683	N−H wagging vibration
645	647	C−C skeletal vibration (sulfonatophenyl radical)
624	618	N−H wagging vibration
581	576	S−O wagging vibration

**Table 5:** Toxicity predictions for TPPS4, using admeSAR pkCSM and ProTox-II webservices.

pkCSM		
Model	Predicted Values	Unit
Water solubility	−2.892	log mol/L
Hepatotoxicity	No	Yes/No
admeSAR		
Model	Results	Probability
Subcellular localization	Lysosome	0.4395
Model	Predicted values	Unit
Aqueous solubility	−3.3743	LogS
Rat Acute Toxicity	2.4748	LD50, mol/kg
ProTox-II		
Model	Predicted values	Unit
Predicted LD50	3066	mg/kg
Predicted Toxicity Class	5	1-bad 6-good
Model	Results	Probability
Hepatotoxicity	Inactive	0.65
Carcinogenicity	Inactive	0.68
Immunotoxicity	Inactive	0.96
Mutagenicity	Inactive	0.62
Cytotoxicity	Inactive	0.75
Aryl hydrocarbon Receptor (AhR)	Inactive	0.78
Mitochondrial Membrane Potential	Inactive	0.85
Phosphoprotein p53	Inactive	0.86
ATPase family AAA domain-containing protein 5 (ATAD5)	Inactive	0.94

**Table 6:** Predicted lowest free energy of binding expressed in kcal/mol and inhibition constant (K_I_) expressed in nanomolar (nM) for pro-caspase 3 and caspase 3.

Target	Estimated Lowest Free Energy of Binding (kcal/mol)	K_I_ (nM)
Pro-caspase 3	−9.31	150.72
Caspase 3	−7.74	2120

## References

[1] O’Neill C.H., Scoggins C.R. (2019). Melanoma. J. Surg. Oncol..

[2] Tripp M.K., Watson M., Balk S.J., Swetter S.M., Gershenwald J.E. (2016). State of the Science on Prevention and Screening to Reduce Melanoma Incidence and Mortality: The Time Is Now. CA Cancer J. Clin..

[3] Davis L.E., Shalin S.C., Tackett A.J. (2019). Current State of Melanoma Diagnosis and Treatment. Cancer Biol. Ther..

[4] Williams P.F., Olsen C.M., Hayward N.K., Whiteman D.C. (2011). Melanocortin 1 Receptor and Risk of Cutaneous Melanoma: A Meta-Analysis and Estimates of Population Burden. Int. J. Cancer.

[5] Seiberg M. (2001). Keratinocyte–Melanocyte Interactions during Melanosome Transfer. Pigment Cell Res..

[6] Leonardi G.C., Falzone L., Salemi R., Zanghì A., Spandidos D.A., McCubrey J.A., Candido S., Libra M. (2018). Cutaneous Melanoma: From Pathogenesis to Therapy (Review). Int. J. Oncol..

[7] Lugović-Mihić L., Ćesić D., Vuković P., Novak-Bilić G., Šitum M., Špoljar S. (2019). Melanoma Development: Current Knowledge on Melanoma Pathogenesis. Acta Derm. Croat..

[8] Domingues B., Lopes J.M., Soares P., Pópulo H. (2018). Melanoma treatment in review. Immunotargets Ther..

[9] Allison R.R. (2014). Photodynamic Therapy: Oncologic Horizons. Future Oncol..

[10] Luo D., Carter K.A., Miranda D., Lovell J.F. (2017). Chemophototherapy: An Emerging Treatment Option for Solid Tumors. Adv. Sci..

[11] Kwiatkowski S., Knap B., Przystupski D., Saczko J., Kędzierska E., Knap-Czop K., Kotlińska J., Michel O., Kotowski K., Kulbacka J. (2018). Photodynamic Therapy—Mechanisms, Photosensitizers and Combinations. Biomed. Pharmacother..

[12] Allison R.R., Downie G.H., Cuenca R., Hu X.-H., Childs C.J., Sibata C.H. (2004). Photosensitizers in Clinical PDT. Photodiagn. Photodyn. Ther..

[13] Allison R.R., Sibata C.H. (2010). Oncologic Photodynamic Therapy Photosensitizers: A Clinical Review. Photodiagn. Photodyn. Ther..

[14] Detty M.R., Gibson S.L., Wagner S.J. (2004). Current Clinical and Preclinical Photosensitizers for Use in Photodynamic Therapy. J. Med. Chem..

[15] Ptaszek M. (2013). Rational Design of Fluorophores for In Vivo Applications. Progress in Molecular Biology and Translational Science.

[16] Triesscheijn M., Ruevekamp M., Aalders M., Baas P., Stewart F.A. (2005). Outcome of MTHPC Mediated Photodynamic Therapy Is Primarily Determined by the Vascular Response. Photochem. Photobiol..

[17] Veenhuizen R., Oppelaar H., Ruevekamp M., Schellens J., Dalesio O., Stewart F. (1997). Does Tumour Uptake of Foscan Determine PDT Efficacy?. Int. J. Cancer.

[18] Chen B., de Witte P.A. (2000). Photodynamic Therapy Efficacy and Tissue Distribution of Hypericin in a Mouse P388 Lymphoma Tumor Model. Cancer Lett..

[19] Cramers P., Ruevekamp M., Oppelaar H., Dalesio O., Baas P., Stewart F.A. (2003). Foscan Uptake and Tissue Distribution in Relation to Photodynamic Efficacy. Br. J. Cancer.

[20] Gomer C.J., Ferrario A. (1990). Tissue Distribution and Photosensitizing Properties of Mono-L-Aspartyl Chlorin E6 in a Mouse Tumor Model. Cancer Res..

[21] Koudinova N.V., Pinthus J.H., Brandis A., Brenner O., Bendel P., Ramon J., Eshhar Z., Scherz A., Salomon Y. (2003). Photodynamic Therapy with Pd-Bacteriopheophorbide (TOOKAD): Successful in Vivo Treatment of Human Prostatic Small Cell Carcinoma Xenografts. Int. J. Cancer.

[22] Schreiber S., Gross S., Brandis A., Harmelin A., Rosenbach-Belkin V., Scherz A., Salomon Y. (2002). Local Photodynamic Therapy (PDT) of Rat C6 Glioma Xenografts with Pd-Bacteriopheophorbide Leads to Decreased Metastases and Increase of Animal Cure Compared with Surgery. Int. J. Cancer.

[23] Seabra A.B. (2017). Iron Oxide Magnetic Nanoparticles in Photodynamic Therapy: A Promising Approach Against Tumor Cells. Metal Nanoparticles in Pharma.

[24] Walker M.G., Jarman P.J., Gill M.R., Tian X., Ahmad H., Reddy P.A.N., McKenzie L., Weinstein J.A., Meijer A.J.H.M., Battaglia G. (2016). A Self-Assembled Metallomacrocycle Singlet Oxygen Sensitizer for Photodynamic Therapy. Chemistry.

[25] Lanzilotto A., Kyropoulou M., Constable E.C., Housecroft C.E., Meier W.P., Palivan C.G. (2018). Porphyrin-Polymer Nanocompartments: Singlet Oxygen Generation and Antimicrobial Activity. J. Biol. Inorg. Chem..

[26] Penon O., Marín M.J., Amabilino D.B., Russell D.A., Pérez-García L. (2016). Iron Oxide Nanoparticles Functionalized with Novel Hydrophobic and Hydrophilic Porphyrins as Potential Agents for Photodynamic Therapy. J. Colloid Interface Sci..

[27] Bechet D., Couleaud P., Frochot C., Viriot M.-L., Guillemin F., Barberi-Heyob M. (2008). Nanoparticles as Vehicles for Delivery of Photodynamic Therapy Agents. Trends Biotechnol..

[28] Zhao C., Ur Rehman F., Yang Y., Li X., Zhang D., Jiang H., Selke M., Wang X., Liu C. (2015). Bio-Imaging and Photodynamic Therapy with Tetra Sulphonatophenyl Porphyrin (TSPP)-TiO2 Nanowhiskers: New Approaches in Rheumatoid Arthritis Theranostics. Sci. Rep..

[29] Morjan I., Alexandrescu R., Dumitrache F., Birjega R., Fleaca C., Soare I., Luculescu C.R., Filoti G., Kuncer V., Vekas L. (2010). Iron Oxide-Based Nanoparticles with Different Mean Sizes Obtained by the Laser Pyrolysis: Structural and Magnetic Properties. J. Nanosci. Nanotechnol..

[30] Nastasa V., Pascu A., Boni M., Smarandache A., Staicu A., Pascu M.L. (2016). Insights into the Photophysics of Zinc Phthalocyanine and Photogenerated Singlet Oxygen in DMSO-Water Mixture. Colloids Surf. A Physicochem. Eng. Asp..

[31] Staicu A., Pascu A., Boni M., Pascu M.L., Enescu M. (2013). Photophysical Study of Zn Phthalocyanine in Binary Solvent Mixtures. J. Mol. Struct..

[32] Rawson J., Angiolillo P.J., Therien M.J. (2015). Extreme Electron Polaron Spatial Delocalization in π-Conjugated Materials. Proc. Natl. Acad. Sci. USA.

[33] Thomsen N.D., Koerber J.T., Wells J.A. (2013). Structural Snapshots Reveal Distinct Mechanisms of Procaspase-3 and -7 Activation. Proc. Natl. Acad. Sci. USA.

[34] Du J.-Q., Wu J., Zhang H.-J., Zhang Y.-H., Qiu B.-Y., Wu F., Chen Y.-H., Li J.-Y., Nan F.-J., Ding J.-P. (2008). Isoquinoline-1,3,4-Trione Derivatives Inactivate Caspase-3 by Generation of Reactive Oxygen Species. J. Biol. Chem..

[35] Berman H.M. (2000). The Protein Data Bank. Nucleic Acids Res..

[36] Pires D.E.V., Blundell T.L., Ascher D.B. (2015). PkCSM: Predicting Small-Molecule Pharmacokinetic and Toxicity Properties Using Graph-Based Signatures. J. Med. Chem..

[37] Cheng F., Li W., Zhou Y., Shen J., Wu Z., Liu G., Lee P.W., Tang Y. (2012). AdmetSAR: A Comprehensive Source and Free Tool for Assessment of Chemical ADMET Properties. J. Chem. Inf. Model..

[38] Banerjee P., Eckert A.O., Schrey A.K., Preissner R. (2018). ProTox-II: A Webserver for the Prediction of Toxicity of Chemicals. Nucleic Acids Res..

[39] Morris G.M., Huey R., Lindstrom W., Sanner M.F., Belew R.K., Goodsell D.S., Olson A.J. (2009). AutoDock4 and AutoDockTools4: Automated Docking with Selective Receptor Flexibility. J. Comput. Chem..

[40] Nistorescu S., Gradisteanu Pircalabioru G., Udrea A.-M., Simon A., Pascu M.L., Chifiriuc M.-C. (2020). Laser-Irradiated Chlorpromazine as a Potent Anti-Biofilm Agent for Coating of Biomedical Devices. Coatings.

[41] Tozar T., Santos Costa S., Udrea A.-M., Nastasa V., Couto I., Viveiros M., Pascu M.L., Romanitan M.O. (2020). Anti-Staphylococcal Activity and Mode of Action of Thioridazine Photoproducts. Sci. Rep..

[42] Bera K., Maiti S., Maity M., Mandal C., Maiti N.C. (2018). Porphyrin–Gold Nanomaterial for Efficient Drug Delivery to Cancerous Cells. ACS Omega.

[43] Zhang Y.-H., Chen D.-M., He T., Liu F.-C. (2003). Raman and Infrared Spectral Study of Meso-Sulfonatophenyl Substituted Porphyrins (TPPSn, N = 1, 2A, 2O, 3, 4). Spectrochim. Acta A Mol. Biomol. Spectrosc..

[44] Aydin M. (2014). Comparative Study of the Structural and Vibroelectronic Properties of Porphyrin and Its Derivatives. Molecules.

[45] Lewandowska K., Rosiak N., Bogucki A., CieleckaPiontek J. (2019). Tuning Electronic and Magnetic Properties in Graphene Oxide—Porphyrins Complexes.

[46] Durães L., Costa B.F.O., Vasques J., Campos J., Portugal A. (2005). Phase Investigation of As-Prepared Iron Oxide/Hydroxide Produced by Sol–Gel Synthesis. Mater. Lett..

[47] Glisenti A. (1998). Interaction of Formic Acid with Fe_2_O_3_ Powders under Different Atmospheres: An XPS and FTIR Study. Faraday Trans..

[48] Coessens V., Schacht E., Domurado D. (1996). Synthesis of Polyglutamine and Dextran Conjugates of Streptomycin with an Acid-Sensitive Drug-Carrier Linkage. J. Control. Release.

[49] Lewandowska K., Rosiak N., Bogucki A., Cielecka-Piontek J., Mizera M., Bednarski W., Suchecki M., Szaciłowski K. (2019). Supramolecular Complexes of Graphene Oxide with Porphyrins: An Interplay between Electronic and Magnetic Properties. Molecules.

[50] DeRosa M.C., Crutchley R.J. (2002). Photosensitized Singlet Oxygen and Its Applications. Coord. Chem. Rev..

[51] Stevens E.A., Mezrich J.D., Bradfield C.A. (2009). The Aryl Hydrocarbon Receptor: A Perspective on Potential Roles in the Immune System. Immunology.

[52] Gottlieb E., Armour S.M., Harris M.H., Thompson C.B. (2003). Mitochondrial Membrane Potential Regulates Matrix Configuration and Cytochrome c Release during Apoptosis. Cell Death Differ..

[53] Udrea A.-M., Dinache A., Pagès J.-M., Pirvulescu R.A. (2021). Quinazoline Derivatives Designed as Efflux Pump Inhibitors: Molecular Modeling and Spectroscopic Studies. Molecules.

[54] Udrea A.-M., Avram S., Nistorescu S., Pascu M.-L., Romanitan M.O. (2020). Laser Irradiated Phenothiazines: New Potential Treatment for COVID-19 Explored by Molecular Docking. J. Photochem. Photobiol. B Biol..

[55] Sparsa A., Faucher K., Sol V., Durox H., Boulinguez S., Doffoel-Hantz V., Calliste C.-A., Cook-Moreau J., Krausz P., Sturtz F.G. (2010). Blue Light Is Phototoxic for B16F10 Murine Melanoma and Bovine Endothelial Cell Lines by Direct Cytocidal Effect. Anticancer Res..

[56] Maytin E.V., Kaw U., Ilyas M., Mack J.A., Hu B. (2018). Blue Light versus Red Light for Photodynamic Therapy of Basal Cell Carcinoma in Patients with Gorlin Syndrome: A Bilaterally Controlled Comparison Study. Photodiagn. Photodyn. Ther..

[57] Dougherty T.J., Gomer C.J., Henderson B.W., Jori G., Kessel D., Korbelik M., Moan J., Peng Q. (1998). Photodynamic Therapy. JNCI J. Natl. Cancer Inst..

[58] Akasov R.A., Sholina N.V., Khochenkov D.A., Alova A.V., Gorelkin P.V., Erofeev A.S., Generalova A.N., Khaydukov E.V. (2019). Photodynamic Therapy of Melanoma by Blue-Light Photoactivation of Flavin Mononucleotide. Sci. Rep..

[59] Ash C., Dubec M., Donne K., Bashford T. (2017). Effect of Wavelength and Beam Width on Penetration in Light-Tissue Interaction Using Computational Methods. Lasers Med. Sci..

[60] Popa I.L., Milac A.L., Sima L.E., Alexandru P.R., Pastrama F., Munteanu C.V.A., Negroiu G. (2016). Cross-Talk between Dopachrome Tautomerase and Caveolin-1 Is Melanoma Cell Phenotype-Specific and Potentially Involved in Tumor Progression. J. Biol. Chem..

[61] Martínez-Lorenzo M.J., Anel A., Alava M.A., Piñeiro A., Naval J., Lasierra P., Larrad L. (2004). The Human Melanoma Cell Line MelJuSo Secretes Bioactive FasL and APO2L/TRAIL on the Surface of Microvesicles. Possible Contribution to Tumor Counterattack. Exp. Cell Res..

[62] Zelickson B., Counters J., Coles C., Selim M. (2006). Light Patch: Preliminary Report of a Novel Form of Blue Light Delivery for the Treatment of Actinic Keratosis. Dermatol. Surg..

[63] Ericson M.B., Wennberg A.-M., Larkö O. (2008). Review of Photodynamic Therapy in Actinic Keratosis and Basal Cell Carcinoma. Ther. Clin. Risk Manag..

[64] Tita S.P.S., Perussi J.R. (2001). The Effect of Porphyrins on Normal and Transformed Mouse Cell Lines in the Presence of Visible Light. Braz. J. Med. Biol. Res..

[65] Lin Y., Zhou T., Bai R., Xie Y. (2020). Chemical Approaches for the Enhancement of Porphyrin Skeleton-Based Photodynamic Therapy. J. Enzyme Inhib. Med. Chem..

[66] Malina L., Tomankova K.B., Malohlava J., Jiravova J., Manisova B., Zapletalova J., Kolarova H. (2016). The in Vitro Cytotoxicity of Metal-Complexes of Porphyrin Sensitizer Intended for Photodynamic Therapy. Toxicol. Vitr..

[67] Rybka J.D. (2019). Radiosensitizing Properties of Magnetic Hyperthermia Mediated by Superparamagnetic Iron Oxide Nanoparticles (SPIONs) on Human Cutaneous Melanoma Cell Lines. Rep. Pract. Oncol. Radiother..

[68] Rueda-Gensini L., Cifuentes J., Castellanos M.C., Puentes P.R., Serna J.A., Muñoz-Camargo C., Cruz J.C. (2020). Tailoring Iron Oxide Nanoparticles for Efficient Cellular Internalization and Endosomal Escape. Nanomaterials.

[69] Lee S.H., Park D.J., Yun W.S., Park J.-E., Choi J.S., Key J., Seo Y.J. (2020). Endocytic Trafficking of Polymeric Clustered Superparamagnetic Iron Oxide Nanoparticles in Mesenchymal Stem Cells. J. Control. Release.

[70] Petri-Fink A., Chastellain M., Juillerat-Jeanneret L., Ferrari A., Hofmann H. (2005). Development of Functionalized Superparamagnetic Iron Oxide Nanoparticles for Interaction with Human Cancer Cells. Biomaterials.

[71] Cengelli F., Voinesco F., Juillerat-Jeanneret L. (2010). Interaction of Cationic Ultrasmall Superparamagnetic Iron Oxide Nanoparticles with Human Melanoma Cells. Nanomedicine.

[72] Hung H.-I., Schwartz J.M., Maldonado E.N., Lemasters J.J., Nieminen A.-L. (2013). Mitoferrin-2-Dependent Mitochondrial Iron Uptake Sensitizes Human Head and Neck Squamous Carcinoma Cells to Photodynamic Therapy. J. Biol. Chem..

[73] Forman H.J., Zhang H., Rinna A. (2009). Glutathione: Overview of Its Protective Roles, Measurement, and Biosynthesis. Mol. Asp. Med..

[74] Gaucher C., Boudier A., Bonetti J., Clarot I., Leroy P., Parent M. (2018). Glutathione: Antioxidant Properties Dedicated to Nanotechnologies. Antioxidants.

[75] Kennedy L., Sandhu J.K., Harper M.-E., Cuperlovic-Culf M. (2020). Role of Glutathione in Cancer: From Mechanisms to Therapies. Biomolecules.

[76] Bi H., Dai Y., Yang P., Xu J., Yang D., Gai S., He F., An G., Zhong C., Lin J. (2019). Glutathione and H_2_O_2_ Consumption Promoted Photodynamic and Chemotherapy Based on Biodegradable MnO_2_–Pt@Au_25_ Nanosheets. Chem. Eng. J..

[77] Shen Z., Ma Q., Zhou X., Zhang G., Hao G., Sun Y., Cao J. (2021). Strategies to Improve Photodynamic Therapy Efficacy by Relieving the Tumor Hypoxia Environment. NPG Asia Mater..

[78] Redza-Dutordoir M., Averill-Bates D.A. (2016). Activation of Apoptosis Signalling Pathways by Reactive Oxygen Species. Biochim Biophys. Acta.

[79] Inhibition of NF-ΚB Signaling Pathway Induces Apoptosis and Suppresses Proliferation and Angiogenesis of Human Fibroblast-like Synovial Cells in Rheumatoid Arthritis.—Abstract—Europe PMC. https://europepmc.org/article/pmc/5999456.

[80] Chen J.-J., Gao L.-J., Liu T.-J. (2016). Photodynamic Therapy with a Novel Porphyrin-Based Photosensitizer against Human Gastric Cancer. Oncol. Lett..

[81] Yu S., Wang G., Shi Y., Xu H., Zheng Y., Chen Y. (2020). MCMs in Cancer: Prognostic Potential and Mechanisms. Anal. Cell. Pathol..

[82] Han W., Wu Y.-Z., Zhao X.-Y., Gong Z.-H., Shen G.-L. (2021). Integrative Analysis of Minichromosome Maintenance Proteins and Their Prognostic Significance in Melanoma. Front. Oncol..

[83] Soares C.D., Borges C.F., Sena-Filho M., Almeida O.P., de Stelini R.F., Cintra M.L., Graner E., Zecchin K.G., Jorge J. (2017). Prognostic Significance of Cyclooxygenase 2 and Phosphorylated Akt1 Overexpression in Primary Nonmetastatic and Metastatic Cutaneous Melanomas. Melanoma Res..

[84] Fei L., Xu H. (2018). Role of MCM2–7 Protein Phosphorylation in Human Cancer Cells. Cell Biosci..

[85] Schaefer S., Doktor T.K., Frederiksen S.B., Chea K., Hlavacova M., Bruun G.H., Rabjerg M., Andresen B.S., Dominguez I., Guerra B. (2019). Down-regulation of CK2α correlates with decreased expression levels of DNA replication minichromosome maintenance protein complex (MCM) genes. Sci. Rep..

[86] Noseda M., Niessen K., McLean G., Chang L., Karsan A. (2005). Notch-Dependent Cell Cycle Arrest Is Associated with Downregulation of Minichromosome Maintenance Proteins. Circ. Res..

[87] Arozarena I., Bischof H., Gilby D., Belloni B., Dummer R., Wellbrock C. (2011). In Melanoma, Beta-Catenin Is a Suppressor of Invasion. Oncogene.

[88] Sinnberg T., Menzel M., Ewerth D., Sauer B., Schwarz M., Schaller M., Garbe C., Schittek B. (2011). β-Catenin Signaling Increases during Melanoma Progression and Promotes Tumor Cell Survival and Chemoresistance. PLoS ONE.

[89] Omori E., Matsumoto K., Ninomiya-Tsuji J. (2011). Non-Canonical β-Catenin Degradation Mediates Reactive Oxygen Species-Induced Epidermal Cell Death. Oncogene.

